# Hepatic galectin-3 is associated with lipid droplet area in non-alcoholic steatohepatitis in a new swine model

**DOI:** 10.1038/s41598-022-04971-z

**Published:** 2022-01-19

**Authors:** Luis V. Herrera-Marcos, Roberto Martínez-Beamonte, Manuel Macías-Herranz, Carmen Arnal, Cristina Barranquero, Juan J. Puente-Lanzarote, Sonia Gascón, Tania Herrero-Continente, Gonzalo Gonzalo-Romeo, Víctor Alastrué-Vera, Dolores Gutiérrez-Blázquez, José M. Lou-Bonafonte, Joaquín C. Surra, María J. Rodríguez-Yoldi, Agustín García-Gil, Antonio Güemes, Jesús Osada

**Affiliations:** 1 Departamento de Bioquímica y Biología Molecular y Celular, Facultad de Veterinaria, Instituto de Investigación Sanitaria de Aragón-Universidad de Zaragoza, Miguel Servet, 177, 50013 Zaragoza, Spain; 2 Instituto Agroalimentario de Aragón, CITA-Universidad de Zaragoza, Zaragoza, Spain; 3 Departamento de Patología Animal, Facultad de Veterinaria, Instituto de Investigación Sanitaria de Aragón-Universidad de Zaragoza, Zaragoza, Spain; 4 Servicio de Bioquímica Clínica. Hospital, Clínico Universitario Lozano Blesa, Zaragoza, Spain; 5 Departamento de Farmacología, Fisiología, Medicina Legal y Forense, Instituto de Investigación Sanitaria de Aragón-Universidad de Zaragoza, Zaragoza, Spain; 6 Servicio General de Apoyo a la Investigación. División de Experimentación Animal, Universidad de Zaragoza, Zaragoza, Spain; 7 Ebers Medical Technology, Zaragoza, Spain; 8 Unidad de Proteómica, Universidad Complutense de Madrid, Madrid, Spain; 9 Departamento de Producción Animal y Ciencia de los Alimentos, Escuela Politécnica Superior de Huesca, Instituto de Investigación Sanitaria de Aragón-Universidad de Zaragoza, Huesca, Spain; 10 Departamento de Cirugía, Facultad de Medicina, Instituto de Investigación Sanitaria de Aragón-Universidad de Zaragoza, Zaragoza, Spain; 11 CIBER de Fisiopatología de la Obesidad y Nutrición, Instituto de Salud Carlos III, Madrid, Spain

**Keywords:** Metabolic diseases, Liver diseases

## Abstract

Non-alcoholic fatty liver disease (NAFLD) is currently a growing epidemic disease that can lead to cirrhosis and hepatic cancer when it evolves into non-alcoholic steatohepatitis (NASH), a gap not well understood. To characterize this disease, pigs, considered to be one of the most similar to human experimental animal models, were used. To date, all swine-based settings have been carried out using rare predisposed breeds or long-term experiments. Herein, we fully describe a new experimental swine model for initial and reversible NASH using cross-bred animals fed on a high saturated fat, fructose, cholesterol, cholate, choline and methionine-deficient diet. To gain insight into the hepatic transcriptome that undergoes steatosis and steatohepatitis, we used RNA sequencing. This process significantly up-regulated 976 and down-regulated 209 genes mainly involved in cellular processes. Gene expression changes of 22 selected transcripts were verified by RT-qPCR. Lipid droplet area was positively associated with *CD68, GPNMB, LGALS3*, *SLC51B* and *SPP1,* and negatively with *SQLE* expressions. When these genes were tested in a second experiment of NASH reversion, *LGALS3, SLC51B* and *SPP1* significantly decreased their expression. However, only *LGALS3* was associated with lipid droplet areas. Our results suggest a role for LGALS3 in the transition of NAFLD to NASH.

## Introduction

Non-alcoholic fatty liver disease (NAFLD) is currently the most common liver disease among Western countries with a prevalence of 20–34% in the general population^1^. Some of these patients will develop cirrhosis requiring liver transplantation in the looming future^2^. Therefore, improving our understanding of the pathophysiology and progression of NAFLD is of paramount interest to identify new therapeutics targets^3^.

The liver has a key role as a regulator of lipid metabolism, being responsible for the uptake, biosynthesis and catabolism of fatty acids, their export and relocation to other tissues^4^. An imbalance of one or more of these pathways, in the absence of alcohol abuse, results in accumulation of fat within the liver and the subsequent development of NAFLD^5^. However, the molecular mechanisms underlying the pathological fat store within the liver are not fully elucidated^5,6^.

Histologically, NAFLD is largely categorized into simple steatosis, corresponding to the presence of steatosis without additional liver damage, and nonalcoholic steatohepatitis (NASH) which is thought to be a progressive condition which can lead to advanced fibrosis, cirrhosis, hepatocellular carcinoma and liver failure^7–10^. This classification corresponds to the “two-hit hypothesis”, an initial framework for understanding the pathogenesis of NASH^11^. However, the validity of this oversimplified concept has been questioned^12,13^. Other authors propose a modified “two-hit” hypothesis wherein non-esterified fatty acids (NEFAs) coming from lipolysis or de novo lipogenesis play a direct role in promoting oxidative stress and inflammation-mediated liver injury, whereas the esterification of NEFAs can function as a protective mechanism^14^. Moreover, there is also a “multiple parallel hits” hypothesis, which considers the role of gut microbiota and adipose tissue-derived factors^15^.

Transcriptomics, either by DNA- microarrays or sequencing, offers a new possibility to understand the pathophysiology and progression of NAFLD. Both techniques have been validated in nutraceutical studies^16,17^. RNAseq provides accurate transcript levels and their isoforms^18^. Selected examples of its use in swine include characterization of microRNA in adipose tissue, inflammation-related genes in NASH-induced in Bama minipigs^19,20^, meat quality^21^ or immune response in peripheral or intestinal cells^22,23^.

In the search of molecular mechanisms of NAFLD, pigs offer possibilities that would be inviable in humans regarding availability of biological samples and genetic manipulation^24,25^. In this regard, swine is emerging as a valuable translational model to close the gap between rodents and humans. The pig displays genetic, anatomical and physiological human resemblances^26^ in cardiovascular system, gastrointestinal tract, morphology and physiology of the pancreas, body fat distribution, proportional organ sizes, propensity for sedentary behaviour, fat cell size or metabolic disease progressions^20,27–29^. Swine also offers a variety of genetic backgrounds, classified in two main groups: the modern commercial breeds (e.g. Large White, Pietrain) and the minipigs (e.g. Ossabaw, Göttingen, Yucatan, Bama)^24,28^. The easy access to commercial and diverse (natural or selected) pig populations offers an opportunity to combine different phenotypes for specific research purposes^30^. Therefore, pigs are good models for studying human metabolic diseases in a time-dependent manner^31^. Despite all those advantages, detailed information at the porcine gene and protein expressions is lagging behind in comparison to other organisms^20,29^.

Pig could also be a potential source of livers for xenotransplantation in order to provide a therapeutic solution to meet the increasing demand. In this regard, the pig is considered a suitable donor source for xenogeneic applications^32^ and is already used for obtaining heart valves and acellular matrices^25,28^. Moreover, the elimination of pig endogenous retroviruses^33^ and the partial deletion of the immune system response^34^ will favour this choice. Characterization of the swine liver is, therefore, crucial to achieve successful xenotransplantation and a pressing condition to characterize better biomarkers to establish functionality of steatotic livers. With this aim, we have tested a dietary intervention wherein a reversible NAFLD and initial NASH develops in just 8 weeks with easily accessible commercial breeds. To our knowledge, this is the first report achieving an initial NASH in a commercial breed swine model, and an RNAseq approach has been used to characterize this pathology.

## Results

### Characterization of fast and simple development of NASH in a commercial crossbred swine

**Figure 1:**
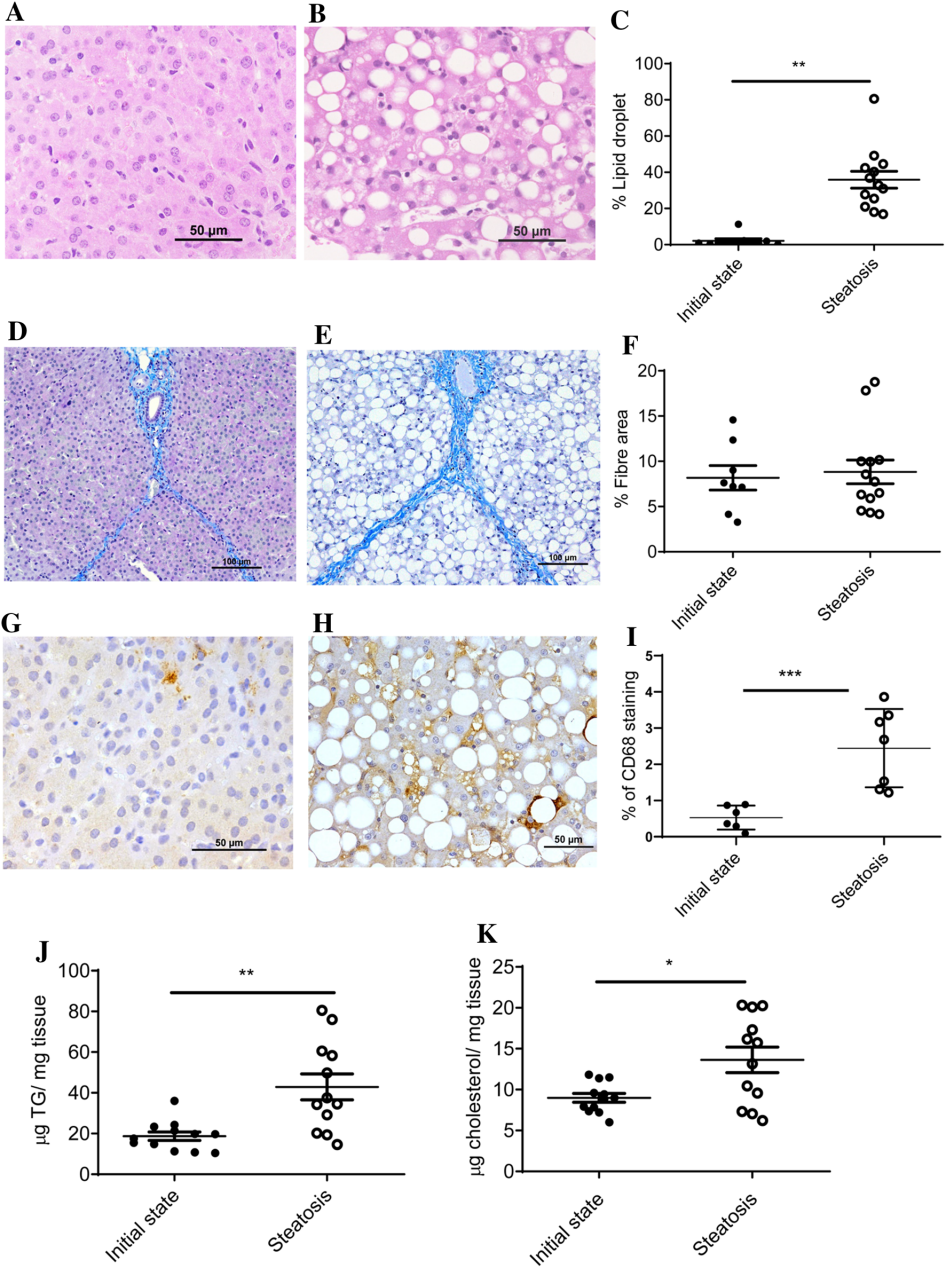
Characterization of liver in a porcine model of dietary NAFLD development. Representative liver micrographs, stained with haematoxylin–eosin, from commercial bred swine before (**A**) and after (**B**) consuming the steatotic diet for 2 months. Morphometric changes in lipid droplet area expressed as percentage of total liver section (**C**). Representative liver micrographs before consuming (**D**) and after consuming the steatotic diet for 2 months (**E**) using Masson’s trichrome staining. Morphometric changes in fibre area (**F**), expressed as percentage of total liver section. Representative CD68 immunostaining from liver sections coming from pigs before consuming (**G**) and after consuming the steatotic diet for 2 months (**H**). Morphometric changes in CD68 positive areas (**I**), expressed as percentage of total liver section. Hepatic triglyceride (**J**) and cholesterol (**K**) contents before and after consuming the steatotic diet for 2 months. Individual values, means and SD are represented for each group. Statistical analyses were carried out using Mann–Whitney U test. *p < 0.05; **p < 0.01.

One of the main problems working with commercial crossbred swine is their genetic resistance to generate NAFLD due to the human directed selection over the last few decades to convert all their intakes into muscle weight gain^35^. Based on our previous experience with medium term experiments^36^, swine fed a high cholate, cholesterol, fructose and saturated fat and methionine- and choline-deficient diet (Table S1) for two months could overcome this drawback for the use of this large animal as a NAFLD model. In fact, the percentage of hepatic area occupied by lipid droplets (Fig. 1A–C and Fig. S1) significantly increased in the pigs consuming this diet for two months without significant changes in fibre areas (Fig. 1D–F). According to CD68 immunostaining, there was an increased number of cells expressing this protein (Fig. 1G–I) suggesting an initial inflammation. Significant increases in hepatic triglyceride and cholesterol (Fig. 1J,K) contents were also observed reinforcing the histological data. Using the NAFLD score^7^ according to Liang et al.^37^, the results were compatible with the development of NAFLD and initial NASH (Table S2). Using fatty liver inhibition of progression (FLIP) algorithm and steatosis, activity, and fibrosis (SAF) score^38^, 17% of pigs consuming the steatotic diet for two months were categorized as NAFLD, 75% of pigs as NASH with score 1 for ballooning and 8% as NASH with ballooning score 2, reinforcing the interpretation that our model of NAFLD may progress to NASH.

Regarding the hepatic-related plasma parameters (Table S3): ALP, GGT and total bilirubin augmented, ALT and AST remained unchanged after the two-month dietary intervention. Other plasma parameters such as glucose, insulin and adiponectin (Table S3) did not increase, indicating the lack of insulin resistance; neither was an open systemic inflammation observed due to their low levels of inflammatory cytokines (even if there were significant increases of IL-6 and IL-8, they remained at a low level). Interestingly, a diminution of an anti-inflammatory cytokine, IL12p40, was observed. Triglycerides (TG), ketone bodies (KB), and NEFA significantly diminished after the dietary treatment. Plasma total cholesterol significantly augmented due to the increase in LDL (Table S3 and Fig. S2). There was a striking increase in plasma leptin by the dietary intervention. Overall, a steatotic model has been developed in a commercial cross-bred swine with a rapid two-month development. Features of the model are hepatic inflammation, absence of insulin resistance and hyperleptinemia.

### Hepatic gene expression in NASH progression study

**Figure 2:**
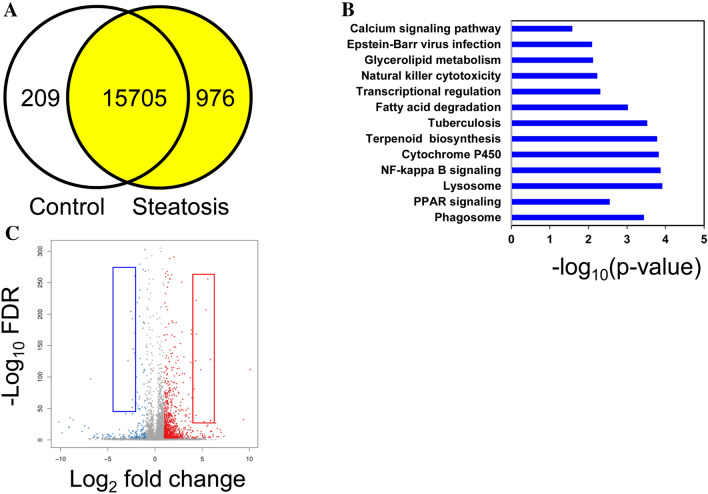
Differentially expressed genes according to RNAseq from livers in a porcine model of dietary NAFLD development. (**A**), Venn diagram analysis showing the transcripts expressed in control (initial state) and steatotic groups (the same animals on the steatotic diet for 2 months) with fold change > 2 and false discovery rate < 0.01. (**B**), Pathway enrichment of differentially expressed genes expressed as the log [–P] analysed by KEGG pathway enrichment. (**C**), Volcano plot representing initial vs. steatotic state differentially expressed genes. Red and blue squares represent the selected genes shown in Supplementary Table S5.

**Figure 3:**
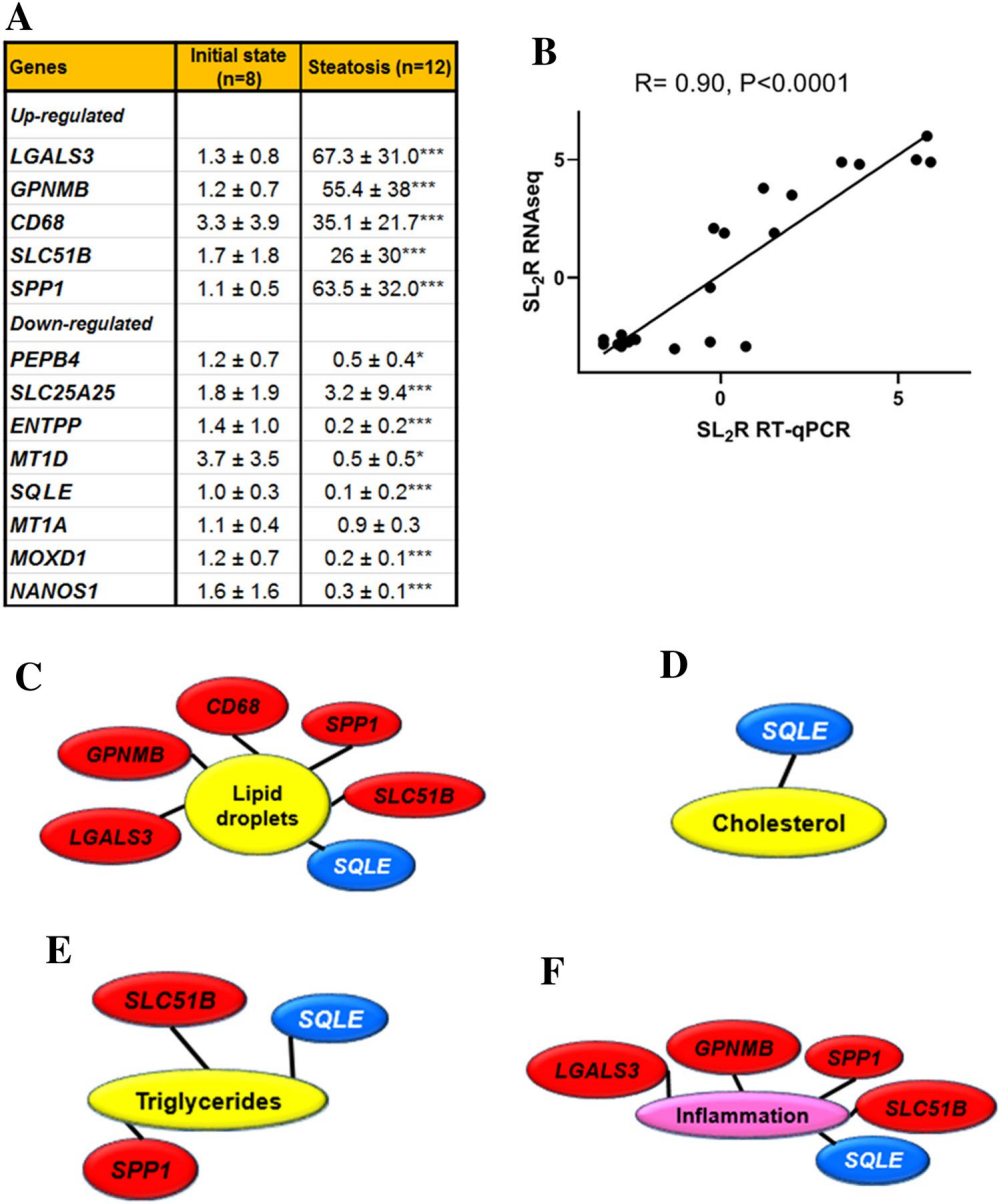
Validation and biological meaning of RNAseq data in a porcine model of dietary NAFLD development. Verification by RT-qPCR of the changes in differentially expressed genes according to RNAseq analysis (**A**). Data (mean ± SD) represent arbitrary units normalized to *UBA52* expression. Statistical analyses were carried out by Mann–Whitney’s U test. *p < 0.05; **p < 0.01 and ***p < 0.005. Correlation analysis between RNAseq and RT-qPCR data (**B**). Log_2_ of steatosis/initial state ratio of RNAseq values of selected genes were plotted against the steatosis/non-steatosis ratio of mean expression values of the same genes by RT-qPCR (see Table S6). Significant (P < 0.001) associations among hepatic lipid droplet content and several gene expressions (**C**), hepatic cholesterol and gene expression (**D**), hepatic triglycerides and gene expressions (**E**) and hepatic inflammation (CD68 immunostaining) and gene expressions (**F**). Red text boxes denote positive associations while blues negative ones. Correlations were calculated according to the Spearman’s rho test**.**

To meet this end, four hepatic RNA pools, each containing an equal amount from two animals, from pigs receiving the control diet and another four prepared from pigs after being fed the steatotic diet were sequenced using next generation sequencing (Table S4). From each library, clean read sequences (46.8 × 10^6^ ± 0.9 × 10^6^), filtered from contaminants, adaptors, low quality regions and reads with unknown bases, were mapped onto reference genome, and followed by gene prediction. In both groups, the mapping ratio was close to 94.0 ± 0.3% for a number of 15,996 known and 305 novel transcripts. Differentially expressed genes, using log_2_ fold change of > 1 and < − 1 and false discovery rate of 0.01, shown in Fig. 2A, were 209 in the control and 976 following the steatotic diet. According to pathway enrichment of differentially expressed genes, Fig. 2B, 13 KEGG pathways were significantly modified. The pathways with more transcripts associated were those dubbed as phagosome (40), tuberculosis (34), Epstein-Barr virus infection (32), transcriptional regulation (30), lysosome (23), calcium signaling pathway (26), PPAR signaling (24) NF-kappa B signaling (22), natural killer cytotoxicity (19), cytochrome P450 (13), fatty acid degradation (11), glycerolipid metabolism (11) and terpenoid biosynthesis (8). A detailed list encompassing all the genes is shown as Table S5. With the exception of lysosome and glycerolipid metabolism pathways, electronic prediction of genes ranged from 13 (terpenoid biosynthesis) to 55% (fatty acid degradation) in function of groups. Figure 2C depicts the volcano plot of distribution of gene expression in function of their significant probability. Supplementary Table S6 lists the most strikingly influenced genes by the steatotic diet (log_2_ fold change < − 2.5 and > 4.5). Based on our previous experience, where only transcripts whose levels were higher than 0.3 fragments per kilo base per million mapped reads (FPKM) and readings displayed in more than 75% of samples, were reliably confirmed by RT-qPCR^39^, a meticulous screening of readings was undertaken. As shown in Fig. S3, some transcripts failed for these criteria, not showing sequences in some control pools, so a new Table 1 reflecting fifteen selected transcripts is shown. Excepting *CD68*, none of these gene expressions was taking into account in the pathway enrichment analyses (Table S5). The genes were sorted into three main categories: cellular processes, metabolism and ion transport. Thirteen differentially expressed genes from the RNAseq data were assayed by RT-qPCR (Fig. 3A). With the exceptions of *SLC25A25* and *MT1A*, all of them showed the same differential expression pattern observed using RNAseq. These 13 transcripts and additional 8 randomly selected (Table S6) were used to compare their magnitude of change expressed as signal log_2_ ratio of both methods. As shown in Fig. 3B, a good agreement was reached by both methods (Pearson’s R = 0.9, P < 0.0001). In our previous experience, not only was the change of the group important, but also the individual expression values^40^. This is particularly relevant in pathological studies where the response of each animal varies in terms of gene expression and disease progression. In order to explore the association of both aspects, hallmarks of NASH such as hepatic steatosis, using its markers such as lipid droplet areas, cholesterol or triglyceride levels, and inflammation, assessed as CD68, were tested with the individual values of gene expression obtained by RT-qPCR by correlation analyses. Lipid droplets were positively (P < 0.001) associated with *CD68, GPNMB, LGALS3*, *SLC51B* and *SPP1,* and negatively with *SQLE* expressions (Fig. 3C). Hepatic cholesterol was inversely associated with *SQLE* expression (Fig. 3D). Hepatic triglycerides were positively associated with *SLC51B* and *SPP1* and negatively with *SQLE* (Fig. 3E)*.* Inflammation, according to CD68 immunohistochemistry, was positively associated with *GPNMB, LGALS3*, *SLC51B* and *SPP1,* and negatively with *SQLE* expressions (Fig. 3F).

**Table 1:** Selected hepatic transcripts differentially upregulated in male pigs fed the steatotic diet according to RNAseq at the level of FDR < 0.001 and FPKM ≥ 0.3.

	Gene ID		Symbol	Initial state (FPKM)	Steatosis (FPKM)	Log_2_ FC
**Up-regulated**						
Apoptosis, cell adhesion	100,038,033	Galectin-3	*LGALS3*	4.3 ± 1.8	274.9 ± 81.9	6.0
Growth delay and reduction of metastatic potential	100,049,669	Glycoprotein NMB	*GPNMB*	24 ± 6	776 ± 303	5.0
Activation of macrophages	100,520,753	Macrosialin	*CD68*	1 ± 0.5	16 ± 9	4.9
Transport of bile acids	100,525,144	Solute carrier family 51 subunit beta	*SLC51B*	0.3 ± 0.1	8.0 ± 4.8	4.8
Production of interferon-gamma and interleukin-12	397,087	Osteopontin	*SPP1*	6.1 ± 2.5	151 ± 73	4.6
**Down-regulated**						
Cu and Zn transport	397,123	Metallothionein-3	*MT3*	8.6 ± 1.6	0.4 ± 0.1	− 4.3
Protein folding	100,514,482	Anterior gradient protein 2	*AGR2*	8 ± 13	0.6 ± 0.1	− 3.7
Resistance to TNF-induced apoptosis	100,302,365	Phosphatidylethanolamine-binding protein 4	*PEBP4*	2.6 ± 0.8	0.3 ± 0.3	− 3.0
Nucleotide import	100,156,347	Calcium-binding mitochondrial carrier protein	*SLC25A25*	64 ± 71	8.3 ± 1.3	− 2.9
Breakdown of phosphoethanolamine	100,519,324	Ethanolamine-phosphate phospho-lyase	*ETNPPL*	23 ± 16	3.2 ± 1.8	− 2.9
Copper transport	100,037,920	Metallothionein-1E	*MT1D*	9491 ± 1350	1318 ± 775	− 2.8
Oxidation of squalene	100,113,409	Squalene epoxidase	*SQLE*	34.6 ± 7	5.1 ± 8.2	− 2.8
Binding of divalent heavy metal ions	102,166,944	Metallothionein-1A	*MT1A*	4311 ± 913	644 ± 325	− 2.7
Copper ion binding	100,151,998	Monooxygenase, DBH-like 1	*MOXD1*	1.8 ± 0.5	0.3 ± 0.1	− 2.7
Post-transcriptional repressor	100,157,783	Nanos C2HC-type zinc finger 1	*NANOS1*	1.3 ± 1.1	0.2 ± 0.0	− 2.6

Data are means ± SD. FPKM, fragments per kilo base per million mapped reads; Log_2_ FC, log_2_ fold change steatosis/ initial state. Only genes with counts in more than 75% of samples have been taken into consideration.

Biological function obtained from https://www.genecards.org/.

### Characterization of NAFLD/initial NASH regression in a new commercial cross-bred swine

**Figure 4:**
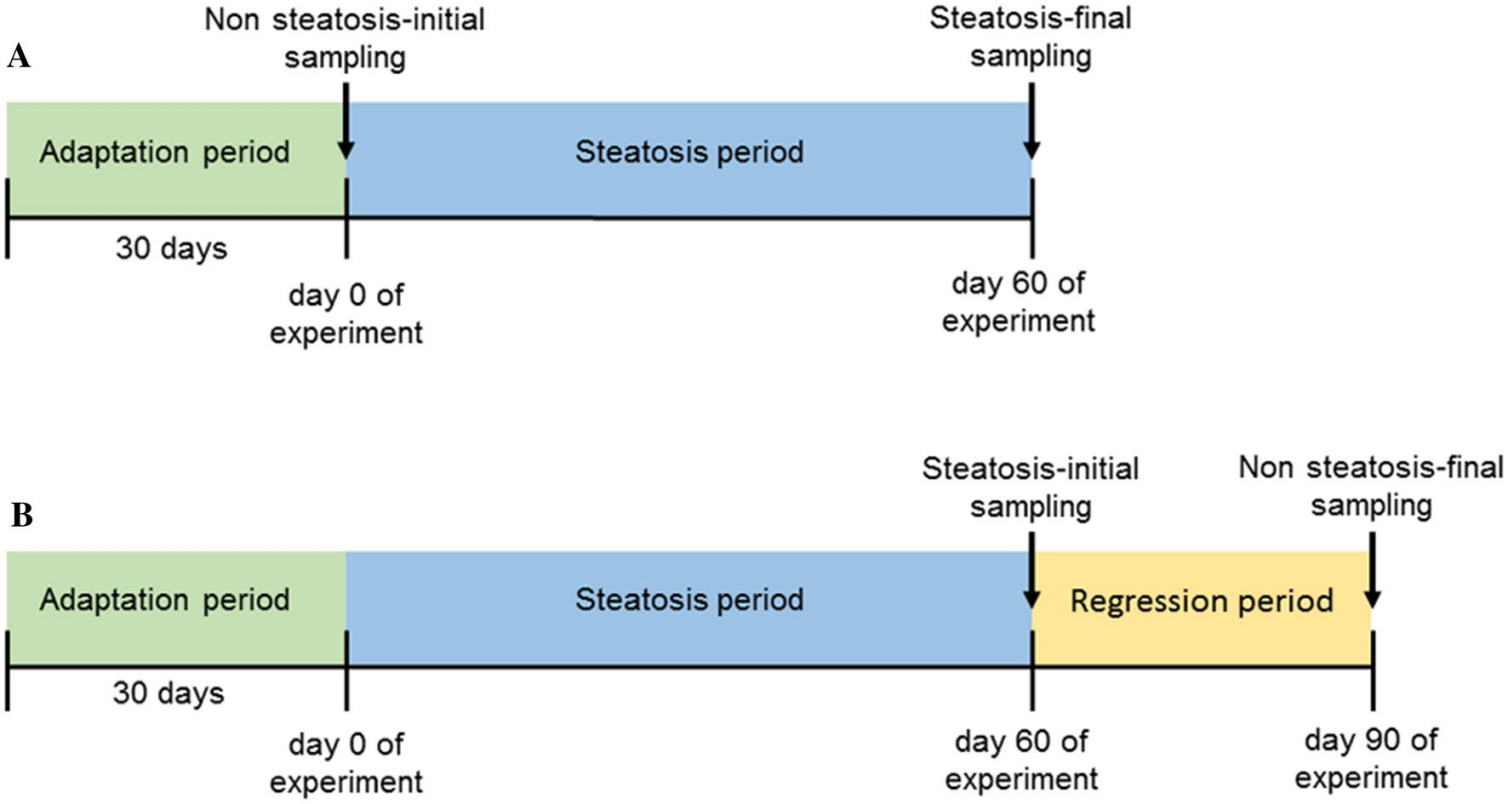
Scheme displaying the used experimental approaches. NAFLD progression study design (**A**) and NAFLD regression experimental setting (**B**).

**Figure 5:**
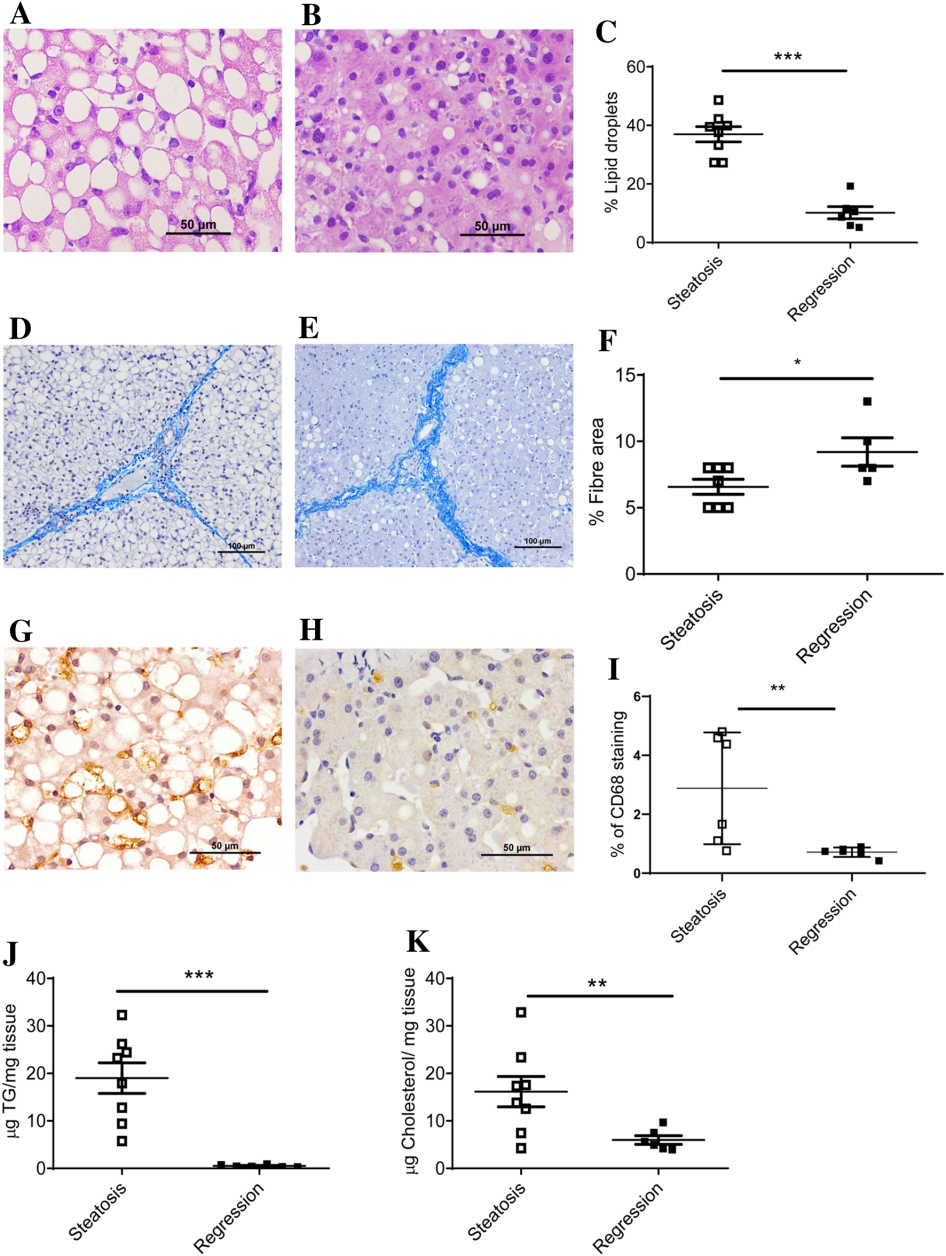
Characterization of NAFLD regression in the porcine model. Representative liver micrographs, stained with hematoxylin–eosin, from swine fed the steatotic diet for two months (**A**) and from the same pigs switched to the control diet for 1 month (**B**). Bar denotes 50 μm. Morphometric changes in hepatic lipid droplet expressed as percentage of area of total liver section (**C**). Representative liver micrographs using Masson’s trichrome staining from swine fed the steatotic diet for two months (**D**) and from the same pigs switched to the control diet for 1 month (**E**). Bar denotes 100 μm. Morphometric changes in fibre extent (**F**) of livers, expressed as percentage of fibre area of total liver section. Representative hepatic CD68 immunostaining from pigs consuming the steatotic diet for two months (**G**) and from the same pigs switched to the control diet for 1 month (**H**). Bar denotes 50 μm. Morphometric changes in CD68 positive areas (**I**), expressed as percentage of total liver section. Hepatic triglyceride (**J**) and cholesterol (**K**) contents from swine fed the steatotic diet for two months and after consuming the control diet for one month. Data are expressed as individual data with means ± SD for each group. Statistical analyses were carried out using Mann–Whitney’s U test. *p < 0.05, **p < 0.01 and ***p < 0.001.

In order to verify whether the present model was able to revert, pigs once done steatotic were switched to their control diet for one month and samples at the end of both analysed phases (Fig. 4). After the pigs received the control diet, hepatic lipid droplets were significantly decreased (Fig. 5A–C) as well as hepatic triglycerides and cholesterol (Fig. 5J,K). Hepatic fibre area was increased (Fig. 5D–F) and inflammation, according to CD68 staining, was significantly reduced (Fig. 5G–I). Total NAS score was significantly decreased (Table S2). In this second experiment, according to the FLIP algorithm and SAF score^38^, 100% of animals after consuming the steatotic diet for two months developed NASH with equal distribution of scores 1 and 2 for ballooning. After switching to the normal diet, 33% of animals reverted their categorization to mild NAFLD, and NASH with score 2 for ballooning was only present in 17% of pigs. There was no change in percentage of animals showing NASH with score 1 for ballooning. Regarding plasma parameters (Table S3), cholesterol, LDL-cholesterol, NEFA, AST, ALT, ALP, GGT, total bilirubin and insulin significantly decreased when pigs consumed the control diet. The opposite was observed for leptin and IL12p40 levels. Collectively, most findings support a trend to revert to the initial phenotype once the steatotic diet was removed.

### Gene expression in initial NASH regression study

**Figure 6:**
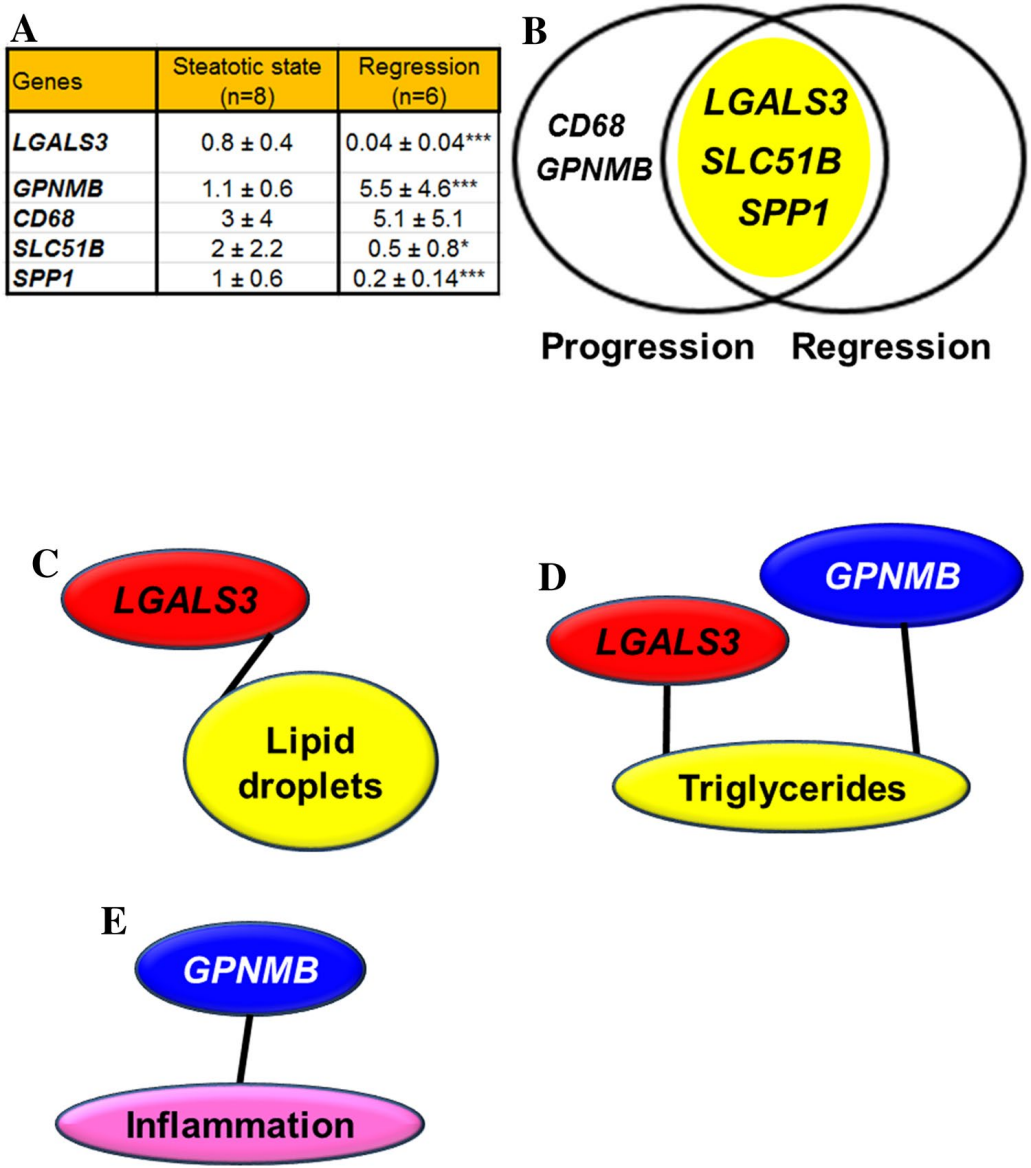
Confirmation of mRNA gene expression changes in the regression experiment using the porcine model of dietary NAFLD development. Analyses by RT-qPCR of previously observed differentially expressed genes (**A**). Data (mean ± SD) represent arbitrary units normalized to *UBA52* expression. Statistical analyses were carried out by Mann–Whitney U test. *p < 0.05, **p < 0.01 and ***p < 0.005. Venn diagram analysis showing the transcripts displaying increased expression in the steatotic condition (yellow area) of progression and regression experiments (**B**). Significant (P < 0.001) association between hepatic lipid droplet content and *LGALS3* expression (**C**), hepatic triglycerides and gene expressions (**D**) and hepatic inflammation (CD68 immunostaining) and gene expressions (**E**). Red colour denotes a positive association while blue a negative one. Correlations were calculated according to the Spearman’s rho test**.**

This model was used to verify whether the observed genetic expression changes were consistent with the decrease in steatosis. To this end, only those genes differentially expressed and significantly associated with hepatic lipid droplets, triglycerides or inflammation in the first experiment were assayed. As shown in Fig. 6A, *LGALS3*, *SLC51B* and *SPP1* significantly decreased their expression while *GPNMB* increased and *CD68* did not change. *LGALS3*, *SLC51B* and *SPP1* expression were in close association with the outcome of hepatic steatosis in both experimental approaches (Fig. 6B). Once again, the significant in-group changes were verified at the individual level by correlation analyses. Only, *LGALS3* expression was significantly associated with lipid droplet areas (Fig. 6C). Hepatic triglycerides were also positively associated with *LGALS3* and negatively with *GPNMB* expressions (Fig. 6D) and inflammation was negatively associated with *GPNMB* expression (Fig. 6E).

### Confirmation at protein level of RNA changes

**Figure 7:**
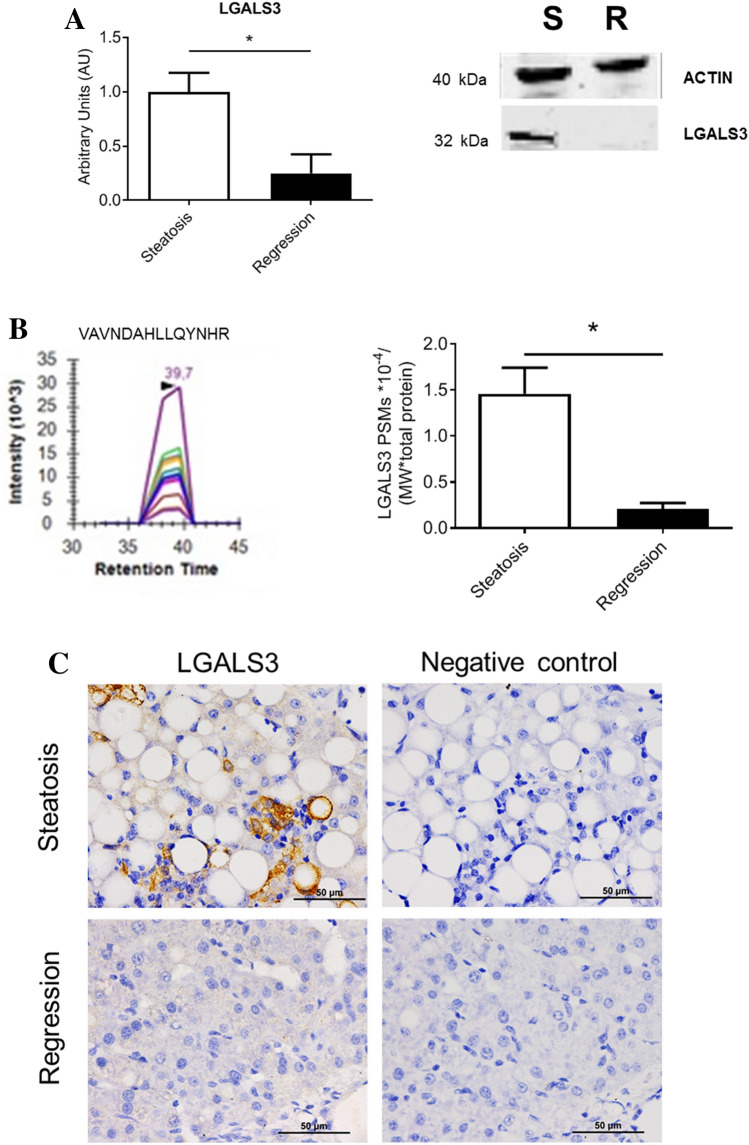
Confirmation of selected protein expression changes in the regression experiment using the porcine model of dietary NAFLD development. Representative Western blots (extracted from original, Supplemental Fig. S2) of proteins and quantification of their expressions normalized to the ACTIN (40 kDa) as loading control (**A**). Parallel reaction monitoring detection of proteotypic peptides and quantification of detectable proteins in targeted proteomics (**B**). Representative chromatograms of proteotypic peptide (VAVNDAHLLQYNHR for LGALS3). PSMs mascot values were divided by their protein molecular weight and the number of detected proteins of each sample. Data are expressed as mean ± SD for each group. Statistical analyses were carried out using Mann–Whitney U test. *p < 0.05. Representative micrographs of immunohistochemical localization of LGALS3 in the liver (**C**). Positive signals of immune labelling are shown in brown. Bar denotes 50 μm.

Due to the striking and consistent changes in *LGALS3* expression, both at the group and individual levels in both experiments, and its strong association with lipid droplet area, hepatic LGALS3 protein levels were assayed by Western blot (Fig. 7A and Fig. S4) and significant changes were observed in agreement with their RNA findings. In order to corroborate the Western blot results, mass-spectrometry was carried out using at least three proteotypic peptides (Fig. 7B). The obtained results also corroborated those found by Western blot. Likewise, immunohistochemistry analysis was in agreement with these previous findings. LGALS3 that showed a cytoplasmic distribution, tended to cluster and displayed an irregular pattern and was almost inexistent in regression livers (Fig. 7C).

## Discussion

This study shows an experimental model of NAFLD with initial NASH in a large animal such as the commercial bred pig through altering its diet for a short period. The diet manipulation involved a combination of high cholate, cholesterol, fructose and saturated fat, and low methionine and choline contents (Table S1). This diet was able to develop a reproducible model of hepatic steatosis with initial NASH in two months. Our biochemical characterization proved that this model showed hypercholesterolemia, hypotriglyceridemia, low level of systemic inflammation, absence of insulin resistance and variable leptinemia (Table S3). The hepatic lipid accumulation and inflammation were reverted when the steatotic diet was removed for a month, but not the fibre extent (Figs. 1 and 5). Using RNAseq to assess hepatic transcriptome, several changes in gene expression were identified and confirmed by RT-qPCR. Lipid droplets were positively associated with *CD68, GPNMB, LGALS3*, *SLC51B* and *SPP1* expressions (Fig. 3). Only, *LGALS3* expression was associated with lipid droplets area in the reversion experiment (Fig. 6). The mRNA changes were also observed at the protein level (Fig. 7). Our results in this experimental design could suggest a potential role for LGALS3 in the NASH development.

The availability of a large animal model of NAFLD with initial NASH in a short period may be of particular interest to understand the onset of NAFLD and its progression to NASH, to explore new treatments for this ailment^41^ and to better characterize the steatotic liver for transplant procedures^36^. In these two experiments, 20 animals were provided the steatotic diet and all of them reached NAFLD according FLIP algorithm. However, only an 8% of animals in the first experiment developed NASH with score 2 of ballooning and 17% only developed NAFLD. In the second experiment, using pigs more genetically defined through artificial insemination with only one boar as progenitor, 100% of pigs showed the NASH condition with equal distribution of scores 1 and 2 for ballooning. These allow us to assume that this steatotic intervention in these animals allows the progression of NAFLD to NASH and the penetrance of the phenotype changes by the genetic repertoire of some individuals. Changes in expression levels in such variable genetic backgrounds represent a unique opportunity to discover new genes involved in steatosis and initial NASH.

Using dietary manipulation, Ossabaw miniature pigs have been proposed as a NASH model in large animals^42,43^. They also observed that metabolic syndrome induced by dietary fructose in the absence of dyslipidemia was not sufficient to cause liver injury. Our data in Large White-Landrace pigs also indicate that dyslipidemia in the absence of metabolic syndrome, is crucial for the development of NAFLD and NASH. Therefore, our model without metabolic syndrome resembles the phenotype of NASH observed in some non-obese subjects^44^.

**Figure 8:**
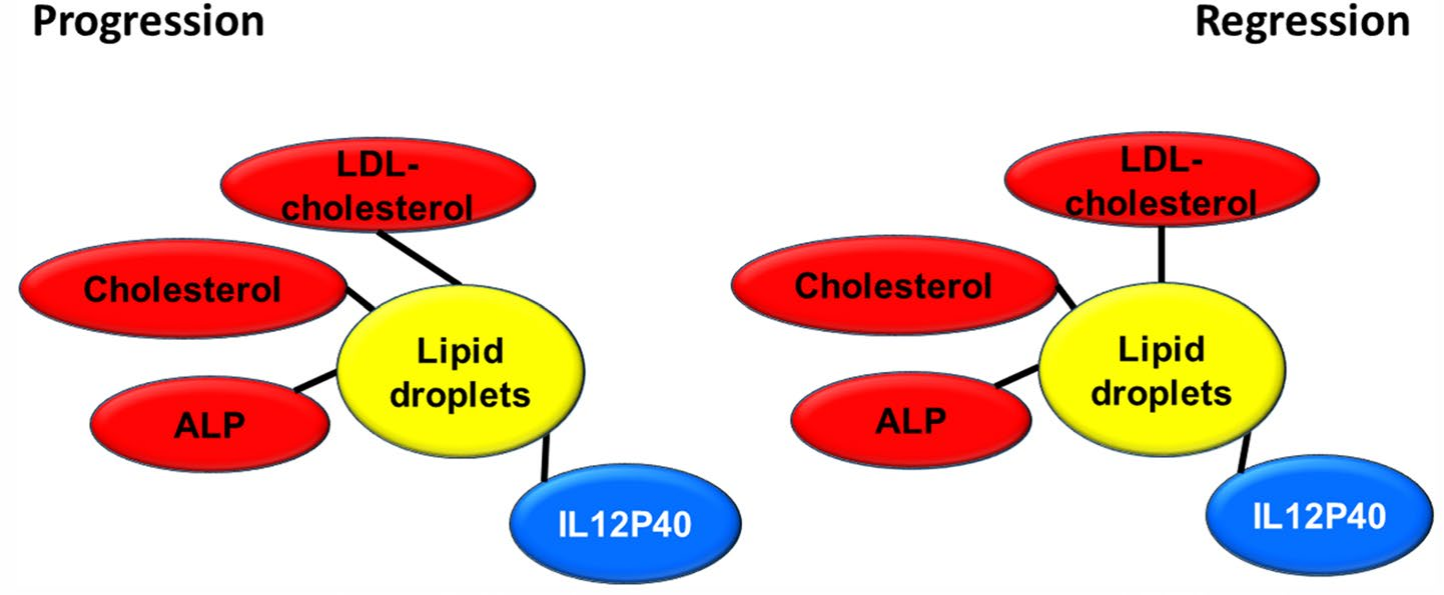
Significant association of plasma parameters and lipid droplet area in both experimental settings. Red colour denotes a positive significant association while blue a negative one. Correlations were calculated according to the Spearman’s rho test**.**

Plasma TG, KB and NEFA were found significantly diminished with the steatosis treatment (Table S3), which could be due to a deficient mobilization of stored fat, decreased use of fat as source of energy or a blockage of TG to be loaded into VLDL^3^. The finding of hypercholesterolemia, mainly carried by LDL (Fig. S2) rejects the latter possibility considering that LDL originate from VLDL^45^. Furthermore, in the second experiment of regression, this new set of pigs showed increased VLDL cholesterol (Fig. S2). Increased total bilirubin, ALP and GGT without changes in aminotransferases recapitulates findings observed in some types of NAFLD patients^46^ and in juvenile Ossabaw swine consuming high-fat, high-fructose, high-cholesterol diet^43^. However, in our case only plasma ALP, total and LDL cholesterol were significantly and positively associated with lipid droplet area in both experimental designs (Fig. 8), while plasma IL12P40 showed a significant negative association. This represents a new aspect not previously reported in humans^47^. In mice, plasma IL12P40 levels were increased after a high fat diet^48^, variable effects according to kaempferol doses^49^ and decreased in the fibrotic liver^50^. Further investigation should be carried out to confirm whether or not this association between this anti-inflammatory protein and NAFLD is consistent among species.

The tenfold increase in leptinemia found in steatosis of the first experiment of progression was not observed in the second regression experiment (Table S3). Several aspects should be considered. The latter experiment was carefully designed by artificial insemination with only one boar as progenitor, so a founder effect cannot be rejected. Moreover, in the regression experiment the steatotic group showed an early peak in the FPLC cholesterol profile compatible with the presence of VLDL or chylomicron remnants (Fig. S2). Fatty acids are known to inhibit leptin secretion from adipocytes^51^, and this effect could have been carried out by VLDLs.

**Figure 9:**
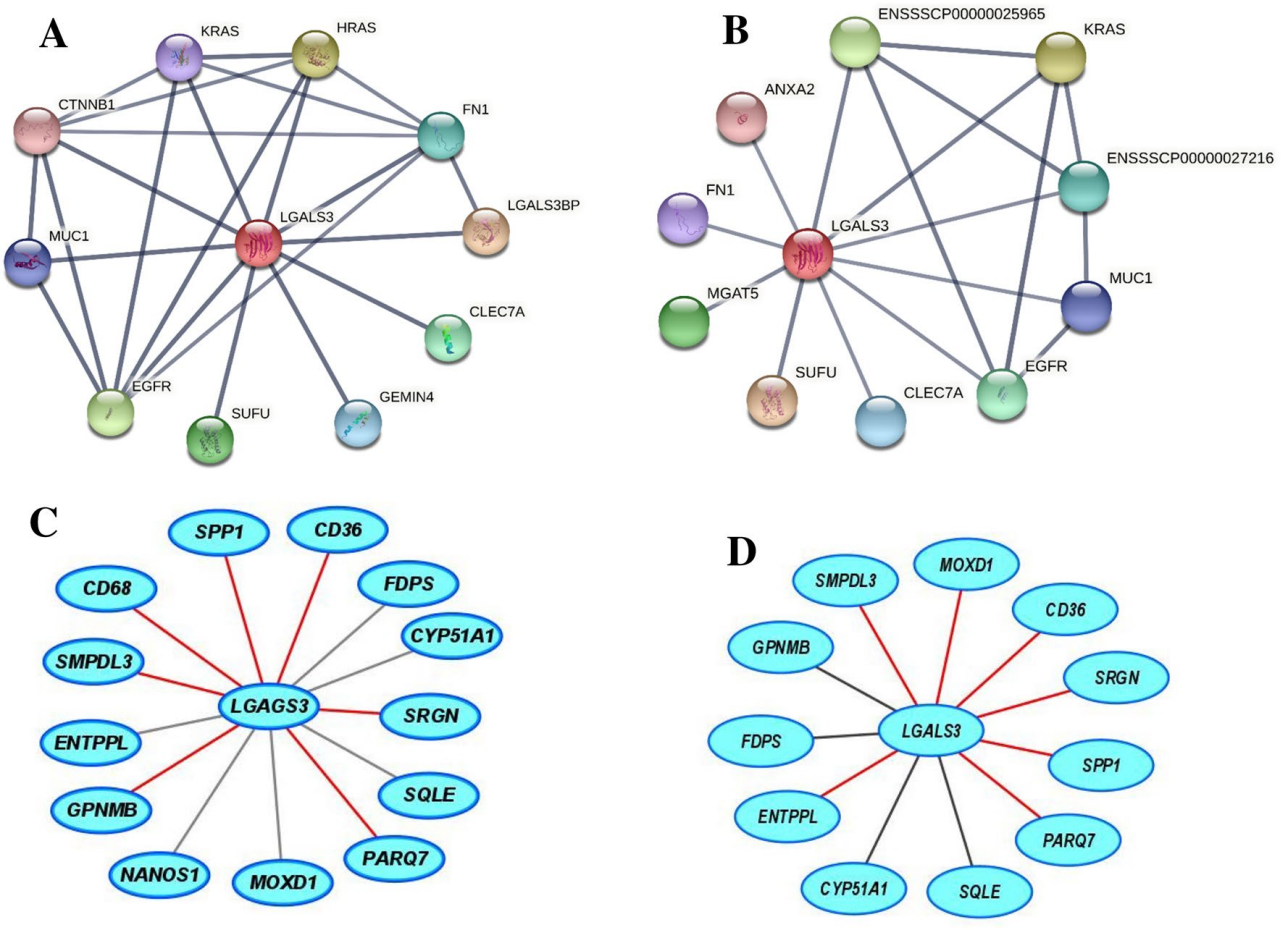
Proposed networks of LGALS3. Human (**A**) and porcine (**B**) LGALS3 networks generated using String (https://string-db.org/). Porcine *LGALS3* gene networks in the progression (**C**) and regression (**D**) experiments. Red colour edges indicate positive significant associations while grey negative ones. Correlations were calculated according to the Spearman’s rho test and Cytoscape 9.0 was used to represent the networks.

Current transcriptomic analyses are carried out using pathway enrichment or the most expressed transcripts. Both aspects have been addressed in the present work. In this sense, our data point out the significant involvement of 13 pathways (Fig. 2B). According to the second strategy (Table 1, Fig. 3A), we have also detected significant changes. The former approach presents important limitations, most of genome function is unknown, it is estimated that 30% of 20,344^52^ or 20,352^53^ human protein-coding genes do not have an assigned function (https://www.encodeproject.org/), and even more, annotation was electronically predicted in most cases. As shown in Table S4, the latter aspect varied from 0 to 55% according to groups. In light of these limitations, there is room for other approaches. In the present report, we used a highly restrictive strategy of gene selection based on striking gene expression changes by RNAseq (readings present in 75% of samples, FPKM > 0.3 and significant top changes according to SL_2_R), their confirmation by RT-PCR in groups. Then, we adopted a new insight analysing how the gene expression of an animal fits with its pathological status represented by hepatic lipid droplet area, cholesterol or triglyceride contents or inflammation, and this were searched by calculation of correlation coefficients. Only transcripts fulfilling these criteria were tested in the regression experiment, and then, their significant group changes and individual associations were verified again (Fig. S5). We successfully used a similar algorithm to find new gene expressions involved in murine steatosis development^40^, and it resulted in two interesting gene expressions: CIDEC*,* involved in sex-specific enlargement of lipid droplets^54^, and SYT1, participating in remodelling of plasma membrane^55^. Interestingly, none of them showed relevant changes in the current experimental design. In the first experiment, *GPNMB, LGALS3*, *SLC51B*, *SPP1* and *SQLE* gene expressions were significantly associated with lipid droplet area and inflammation (Fig. 3C,F). However, only *LGALS3*, *SLC51B* and *SPP1* expressions showed an expected result in the regression experiments. *SLC51B* is a bile acid transporter^56^ and *SPP1* codifies for osteopontin that has been involved in NAFLD^57^. In the present research, only *LGALS3* gene expression changes were correlated with lipid droplet areas in the reversion experiment and emerging as a candidate gene to be activated in the transition of NAFLD into NASH. According to GEO database, further evidences link hepatic *LGALS3* expression and the use of high fat diets in dietary interventions (Table S8). LGALS3 has been related to cardiometabolic disease^58^ and hepatic steatosis^59^. Its gene deletion has been observed to cause both steatosis^60^ and resistance to steatosis in mice^61^. A role for LGALS3 in promoting fibrosis and inflammation in mouse models of nonalcoholic steatohepatitis has been also proposed^59,62^. In the human liver, *LGALS3* expression was found slightly but significantly increased in patients suffering from alcoholic hepatitis and hepatitis B virus-associated acute liver failure and significantly decreased in obese subjects in response to a short-term low-fat hypocaloric diet (Table S9). However, in paediatric NAFLD, the number of galectin-3 positive cells was associated with tissue damage in different ways, suggesting a dual role of this protein^63^. Controversial results have been observed regarding plasma galectin-3, while for some authors it was a good marker of fibrosis in cirrhosis and toxic hepatitis^64^, no association between circulating galectin-3 levels and NAFLD was found in these patients^65^. An inhibitor of galectin-3, GR-MD-02 (belapectin), was safe and well tolerated in phase 1 studies^66^. In phase 2b, this drug was safe but not associated with significant reduction in fibrosis compared with placebo except for a subgroup of patients without oesophageal varices^67^. Since LGALS3, galectin-3, has emerged as a canonical^62^ and noncanonical inflammasome activator in macrophages^68^ and has been found in myeloid-derived cells^23^, it is plausible that activation LGALS3 in Kupffer cells is an early event in the cascade of events taking place in the progression from hepatic simple steatosis into NASH. However, in rats, hepatic stellate cells production of galectin-3 contributes to the expansion of hepatic progenitor cells^69^. Galectin-3 also regulates the capacity of dendritic cells to support killer T-cell-mediated liver injury, playing an important pro-inflammatory role in acute liver injury^70^. Compared with described networts in String data base for human and pig LGALS3, our analysis is providing consistent new interactions of these gene expressions (Fig. 9). A limitation of our study is that no specific mechanism of this protein was pursued. Further experiments will be required to establish the implication of LGALS3 in all these processes.

In conclusion, we present a fast dietary development of a reversible, initial NASH model in a commercial bred swine characterized by hepatic lipid accumulation, inflammation and fibrosis development, neither systemic inflammation nor insulin resistance, but hypercholesterolemia in a short period of time. The model recapitulates some of the features found in human reinforcing the adequacy of this non-murine model and emphasizes that systemic inflammation and insulin resistance are not essential for NAFLD and NASH. Therefore, commercial cross-bred swine can be a model of experimental steatosis evolving into NASH. In our model, ALP, cholesterol, LDL-cholesterol, and IL12P40 are good plasma parameters to study the setting of steatosis progressing to NASH and its reversion among the studied parameters. Further attention should be addressed regarding IL12P40 plasma levels and their implication in NAFLD pathology. Hepatic *LGAL3* expression shows a strong association with lipid droplet area. It is a *bona fide* marker of steatosis evolving into steatohepatitis and its reversion. In this way, it could play an important and unknown role in initial NASH and pathology of pig livers. Further research is warranted to understand its in vivo function and relevance.

## Methods

### Animals and experimental design

Two experimental settings, the progression and the regression of liver steatosis, are the basis of this report. Regarding the first approach (Fig. 4A), after one month of adaptation, twelve male Large White x Landrace pigs, weighing 42 ± 1.5 kg, were used to induce liver steatosis by feeding a steatotic diet for two months and then euthanized. Once completed the analyses of this experiment, the second approach was tackled (Fig. 4B). To this purpose, we generated by artificial insemination a litter of pigs coming from one boar as progenitor (Cooperativa Ganadera de Caspe, Zaragoza, Spain). After one month of adaptation, eight male Large White x Landrace pigs, weighing 38 ± 2.8 kg, were fed the steatotic diet for 2 months. At that moment, they were switched to the control diet for a month and then, euthanized. Unfortunately, only six out of eight animals completed this second experiment.

### Diets

Control diet, purchased from Cadebro (Casetas, Zaragoza, Spain), provided 72.8% of energy from complex carbohydrates, 18% from protein and 9.2% from fat (Supplemental Table S1). The steatotic diet, prepared at the Veterinary School Facility of the University of Zaragoza, was designed to be methionine-deficient and choline-restricted. It was also enriched in 2% cholesterol (Sigma-Aldrich, Germany), 0.5% sodium cholate (Molekula Group, Darlington, UK) and saturated fat (JL Supervía, Cuarte de Huerva, Zaragoza, Spain). This diet provided 50% of energy from hydrogenated palm and sunflower oil (approximatively, saturated fatty acids represented a 50%), 43% from complex carbohydrates and 7% from protein. All animals had unrestricted access to food and water.

### Sampling

As displayed in Fig. 4, samples were obtained at the beginning and at the end of the experiments for both experimental settings after overnight fasting. Sampling was performed under general anaesthesia, induced and maintained with Propofol® (B/Braun-Vetcare, Rubí, Barcelona, Spain) administration. Liver biopsies were obtained by laparotomy. At the end of the experiment, the livers and blood samples were obtained from pigs euthanized by an anaesthetic overdose. Pieces of the livers were stored in 4% paraformaldehyde and some immediately frozen in liquid nitrogen. The frozen livers and plasma were stored at -80ºC. All the procedures were performed according to the European Union guidelines for the handling and care of laboratory animals, in accordance with ARRIVE guidelines and the protocol was approved by the Ethics Committee for Animal Research of the University of Zaragoza (PI43/15).

### Histological analyses

Paraformaldehyde-stored liver samples were embedded in paraffin. Sections (4 μm) were stained with haematoxylin and eosin or Masson’s trichromic technique. Images were captured using a Nikon microscope. Lipid droplet and fibre areas were blindly evaluated as previously described^71^. Morphometric analyses were carried out using Adobe Photoshop CS3 and expressed as percentage of total liver section. NAFLD score (NAS) was assessed using criteria described by Kleiner et al.^7^ with the standardization proposed by Liang et al.^37^. The FLIP algorithm and steatosis, activity, and fibrosis (SAF) score was also used to categorize the histological stage of pigs^38^.

### Hepatic lipid assays

Liver (20 mg) lipids were extracted according to Folch’s method^72^. The organic phases, evaporated under N_2_ stream and dissolved in isopropanol, were used to measure cholesterol and triglyceride contents using Infinity Reagents from Thermo (Thermo Fisher Scientific, Waltham, MA, USA).

### Plasma parameters

Samples were analysed for triglycerides using Infinity Reagent (Thermo) and total, non-esterified, low-density lipoprotein (LDLc) and high-density lipoprotein cholesterol (HDLc) using a fluorometric method (Amplex Red, Molecular Probes, USA). Ketone bodies and non-esterified fatty acids were assayed using kits from Fujifilm (Wako Pure Chemical Corporation, Tokyo, Japan). Biochemistry parameters (glucose, bilirubin, AST, ALP, ALT and GGT) were measured at the Clinical Laboratory of *Hospital Clínico Universitario Lozano Blesa* (Zaragoza, Spain). Insulin (EP0100, Finetest Wuhan, Hubei, China), leptin (EP0103, Finetest) and adiponectin (EP0006, Finetest) were determined by commercial ELISAs following manufacturers’ instructions. Plasma IL-1beta, IL-4, IL-6, IL-8, IL-10, IL-12p40, IFN-alpha, IFN-gamma, and TNF-alpha were assayed using 15-μl sample through a Porcine ProcartaPlex™ assay (EPX090-60829-901, Thermo) according to manufacturer’s instructions.

### RNA preparation

RNA of each liver was extracted using Tri Reagent from Ambion® (Life Technologies, Carlsbad, CA, USA) following the manufacturer’s instructions. DNA contamination was removed using TURBO DNAse treatment and removal kit from Ambion® (Life Technologies). RNA was quantified by absorbance at 260 nm, and purity was verified regarding the A_260/280_ ratio (greater than 1.8) with SPECTROstar Spectrophotometer’s LVis plate (BMG Labtech, Offenburg, Germany). Integrity of the 28S and 18S ribosomal RNAs was verified by 1% agarose gel electrophoresis containing ethidium bromide. Quality of RNAseq samples (RQI > 9) was also verified by Bio-Rad experion chips (Hercules, CA, USA).

### RNAseq analyses

Samples belonging to the first experiment were used for this procedure. Four pools of non steatosis-initial stage were prepared using equal amounts of hepatic total RNA of two pigs. A similar protocol was followed for hepatic total RNA from pigs at the steatotic-final stage. The resulting 8 samples were sent to BGI (Shenzhen, China) service. Their total RNA quality was tested using Agilent 2100 Bioanalyzer (Agilent RNA 6000 nano kit, Santa Clara, CA, USA), and used for library construction. Pair-end reads of 100 bp were read through the BGISEQ-500 platform. Sequencing reads which contained low-quality, adaptor-polluted and high content of unknown base reads were removed before downstream analyses. After filtering, mapping of clean reads to reference genome (Ensembl *Sus scrofa* genome 11.1; www.ensembl.org) was performed using HISAT2 (Hierarchical Indexing for Spliced Alignment of Transcripts) resulting in an average genome mapping rate of 94.34%. After genome mapping, StringTie 1.0.4 was used to reconstruct transcripts^73^, with genome annotation information, novel transcripts were identified by using Cuffcompare (a tool of Cufflinks)^74^ and the coding ability of those new transcripts was predicted using Coding Potential Calculator^75^. RMATS^76^ was used to detect differentially splicing genes between samples. After novel transcript detection, novel coding transcripts were merged with reference transcripts to get a complete reference, then clean reads were mapped to it using Bowtie2^77^. Then gene expression level for each sample was calculated with RSEM^78^. Differentially expressed genes were classified according to KEGG annotation using phyper in R. The complete datasets were deposited in the GEO database with the accession number GSE130924.

### RT-qPCR

In order to verify individual changes of gene expression, 500 ng of total RNA were reverse transcribed using the PrimeScript™ RT Reagent Kit (Takara). PCR real time reactions were performed using 2 × qPCRBIO Sgreen Mix Hi-ROX, from PCR Biosystems (London, UK), according the manufacturer’s instructions, in a ViiA7 Real-TIME PCR System (Life Technologies). The used primers (Supplementary Table 9) were designed using NCBI (National Centre for Biotechnology Information) primer design software and checked by BLAST analysis (NCBI) and Ensembl to verify gene specificity and coverage of transcripts for a specific gene. The relative amount of all mRNAs was calculated using comparative 2^− ΔΔCt^ method using QuantStudio Real Time PCR Software (Thermo Scientific). Three reference genes were assayed, being *UBA52* (XM_013991270.1) the most stable mRNA; therefore, it was selected as the reference (house-keeping) gene.

### Western blotting

A 50-mg sample of liver was homogenized in 500 µl of an 8 M urea/2 M thiourea buffer pH 9 followed by centrifugation for 10 min. Protein concentration was measured by Bio-Rad dye binding assay (Bio-Rad, Madrid, Spain). Protein samples (20 μg) were loaded onto a 16% tricine-SDS-polyacrylamide gel^79^ and electrophoresed for 150 min at 85 V in a Bio-Rad Miniprotean cell (Hercules, CA). Proteins were transferred to PVDF membranes (GE Healthcare, Madrid, Spain) using semidry transfer cell Bio-Rad Trans-Blot SD apparatus with 1.5 mA/cm^2^ membrane 25 min at 20 V. Membranes were blocked with a PBS buffer containing 5% BSA for 1 h at room temperature. Then, they were incubated with the primary antibody, diluted in a PBS buffer containing 2.5% BSA and 1% Tween 20 overnight at 4ºC followed by 2 h at room temperature. Rabbit polyclonal antibodies against LGALS3 (ref. 14979-1-AP) (1/ 2000) from Proteintech (Manchester, UK) and ACTIN (1/3000) from Sigma (St Louis, MO, USA) were used. Membranes were washed three times with a PBS buffer containing 0.1% Tween 20 and incubated for 1 h at room temperature with conjugated goat anti-rabbit IgG (H&L) DyLight 800 secondary antibodies (SA5-35575, Thermo-scientific, diluted 1/200,000). Images were captured using an Odyssey®Clx (LI-COR, Bad Homburg, Germany).

### Targeted proteomics

Proteins extracted as above described were sent to the proteomic facility of the Complutense University (Madrid, Spain). After concentration, digestion and desalting, peptides were subjected to parallel reaction monitoring-mass spectrophotometry (PRM-MS) to detect the protein of interest (tr|A3EX84|A3EX84_PIG) in an EASY-nLC 1000 System coupled to the Q-Exactive HF mass spectrometer (Thermo Scientific). The data acquired in the Q-EXACTIVE HF were analysed using Skyline v 4.1 software and were manually examined to confirm the peptides and transitions that provided accurate detection of peptides/proteins. The MS/MS data were carried out using Proteome Discoverer software v.2.2 (Thermo Scientific) with search engine MASCOT 2.6 (MatrixScience, London, UK) to identify the peptides against in house DataBase with the fasta sequence of targeted protein (8 sequences). Contaminant data Base (247 sequences) and *Sus scrofa* proteome (UP000008227) were downloaded from Uniprot (www.uniprot.org).

### Immunohistochemistry

The antigenic sites were retrieved by incubation of the sections in a boiling citrate buffer (10 mM Tris-sodium citrate, 1.9 mM citric acid, pH 6.0). Next, endogenous peroxidase activity was quenched by incubating with the peroxidase blocking solution (K4010, DAKO Corporation, Carpinteria, CA, USA). Immunohistochemistry was performed using rabbit polyclonal antibodies against LGALS3 (ref. 14979-1-AP, diluted 1/ 200, Proteintech) and mouse anti-CD68 (ref. MCA2317GA, diluted 1/ 1,000, BioRad) in a Tris-buffered saline containing 1% BSA and incubated overnight at 4ºC. Peroxidase signal detection was carried out following the manufacturer’s instructions, EnVision + System-HRP (K4010, DAKO Corporation). To assess the specificity of the immune labelling, control sections were incubated without the primary antibody. Finally, the sections were counterstained with haematoxylin.

### Statistical analyses

Data is shown as means ± SD. Variables not showing normal distribution (according to the Shapiro–Wilk’s test) or homology of variance were analysed using the one-tailed Mann–Whitney’s U test. The Statistical Package for Social Sciences version 15 (SPSS, Chicago, IL, USA) or Prism 5 software for Windows (GraphPad, S. Diego, CA, USA) was used for statistical analyses. Correlations between variables were tested using the Pearson or the Spearman’s correlation tests. Differences were considered significant when P < 0.05.

## Supplementary Information

Supplementary Information.
