# Supplementary material for: Hepatic galectin-3 is associated with lipid droplet area in non-alcoholic steatohepatitis in a new swine model
Source: Sci Rep. 2022 Jan 19;12:1024. doi: 10.1038/s41598-022-04971-z (PMC8770509; doi:10.1038/s41598-022-04971-z)
Supplement: Supplementary file 1 — Supplementary Information. [file 41598_2022_4971_MOESM1_ESM.docx]

**Hepatic galectin-3 is associated with lipid droplet area**

**in non-alcoholic steatohepatitis in a new swine model**

Luis V. Herrera-Marcos, Roberto Martínez-Beamonte, Manuel Macías-Herranz, Carmen Arnal, Cristina Barranquero, Juan J. Puente-Lanzarote, Sonia Gascón, Tania Herrero-Continente, Gonzalo Gonzalo-Romeo, Víctor Alastrué-Vera, Dolores Gutiérrez-Blázquez, José M. Lou-Bonafonte, Joaquín C. Surra, María J. Rodríguez-Yoldi, Agustín García-Gil, Antonio Güemes and Jesús Osada

**Table S1. Diet compositions and fatty acid profiles.**

|  | Control diet | Steatotic diet |
| --- | --- | --- |
| **Average energy intake (kcal/pig/day)** | 2622 ± 219 | 3832 ± 576 |
| **Carbohydrates (%)^a^** | 72.8 | 43.0 |
| **Starch (%)^a^** | 45.0 | 17.2 |
| **Sucrose (%)^a^** | 8.1 | 1.5 |
| **Fructose (%)^a^** | 8.5 | 13.5 |
| **Glucose (%)^a^** | 6.7 | 10.8 |
| **Protein (%)^a^** | 18.0 | 7.0 |
| **Fat (%)^a^** | 9.2 | 50.0 |
| **Fat Source** | Palm oil | Hydrogenated palm and sunflower oil |
| **Caprilic acid (C8:0)^b^** | 0.1 | 0.1 |
| **Capric acid (C10:0)^b^** | 0.2 | 0.04 |
| **Lauric acid (C12:0)^b^** | 3.9 | 0.5 |
| **Miristic acid (C14:0)^b^** | 1.8 | 1.0 |
| **Palmitic acid (C16:0)^b^** | 27 | 39.0 |
| **Palmitoleic acid (C16:1)^b^** | 0.1 | 0.2 |
| **Estearic acid (C18:0)^b^** | 2.9 | 8.7 |
| **Oleic acid (C18:1)^b^** | 28.0 | 32.7^c^ |
| **Linoleic acid (C18:2 n6)^b^** | 32.5 | 12.6^c^ |
| **Linolenic acid (C18:3 n3)^b^** | 2.3 | 0.1 |
| **Arachidic acid (C20:0)^b^** | 0.4 | 0.4 |
| **Gadoleic acid (C20:1)^b^** | 0.4 | 0.14 |
| **Cholesterol (ppm)** | Nil to negligible | 20 000 |
| **Sodium cholate (ppm)** | Nil to negligible | 500 |
| **Methionine (ppm)** | 2160 | 100 |
| **Choline (ppm)** | 90 | 16 |

^a^ Values represent percent of total daily calories

^b^ Fatty acid profile percentage

^c^ Trans isomers observed. Oleic: 4%, linoleic: 0.2%

**Table S2. Characterization of hepatic features following the steatotic diet for 2 months and its removal.**

|  | **Experiment A** | | **Experiment B** | |
| --- | --- | --- | --- | --- |
| Histologic lesion | Initial | Steatosis progression | Steatosis  initial | Regression |
| Microvesicular steatosis | 0 ± 0 | 0 ± 0 | 0.3 ± 0.5 | 0.2 ± 0.4 |
| Macrovesicular steatosis | 0 ± 0 | 1.4 ± 0.5 ^a^ | 1.8 ± 0.5 | 0.7 ± 0.5 ^c^ |
| Hypertrophy | 0 ± 0 | 0 ± 0 | 0 ± 0 | 0 ± 0 |
| Inflammation | 1.0 ± 0.9 | 2.3 ± 1.0 ^b^ | 2.8 ± 0.6 | 2.3 ± 0.8 |
| Total score | 1.0 ± 0.9 | 3.7 ± 1.0 ^a^ | 4.9 ± 1.0 | 3.2 ± 1.2 ^c^ |

Experiment A, pigs fed a standard diet; steatosis progression, when the same pigs were fed the steatotic diet for 2 months. Experiment B steatosis initial, independent group of pigs fed the steatotic for two months and regression, the latter group was changed to the standard diet for 1 month. Data represent score numbers for each histological feature and standard deviation according to Kleiner *et al* (Ref 7) and Liang *et al.* (Ref 37). Statistical analyses were carried out using one-tail Mann Whitney’s U test. Experiment A: ^a^, P < 0.001 and ^b^, P < 0.01. Experiment B: ^c^, P < 0.05.

| **Table S3. Serum biochemistry parameters.** | | | | |
| --- | --- | --- | --- | --- |
|  | Experiment A. Progression | | Experiment B. Regression | |
|  | Non Steatosis-initial stage | Steatosis-final stage | Steatosis-initial stage | Regression-final stage |
|  | (n = 12) | (n = 12) | (n = 8) | (n = 6) |
| Glucose (mg/dL) | 91 ± 9 | 93 ± 20 | 92 ± 22 | 92 ± 13 |
| TG (mg/dL) | 33 ±10 | 24 ± 7^a^ | 22 ± 15 | 26 ± 6 |
| Cholesterol (mg/dL) | 82 ± 10 | 147 ± 62^b^ | 156 ± 107 | 77 ± 7^d^ |
| LDLc (mg/dL) | 23 ± 3 | 90 ± 38^c^ | 64 ± 44 | 29 ± 3^e^ |
| HDLc (mg/dL) | 54 ± 7 | 52 ± 22 | 58 ± 40 | 43 ± 4 |
| Non-esterified cholesterol (mg/dL) | 17 ± 2 | 23 ± 10 | 60 ± 41 | 24 ± 2^e^ |
| KB (µM) | 149 ± 87 | 36 ± 35^c^ | 31 ± 35 | 0 ± 0 |
| NEFA (mM) | 0.11 ± 0.11 | 0.05 ± 0.09^a^ | 0.23 ± 0.35 | 0.37 ± 0.36 |
| AST (IU/L) | 48 ± 16 | 60 ± 22 | 60 ± 24 | 33 ± 23^d^ |
| ALT (IU/L) | 22 ± 3 | 24 ± 5 | 24 ± 4 | 18 ± 2^e^ |
| ALP (IU/L) | 5 ± 2 | 132 ± 74^c^ | 136 ± 84 | 3 ± 1^e^ |
| GGT (IU/L) | 51 ± 13 | 60 ± 10^a^ | 50 ± 10 | 34 ± 5^e^ |
| Total bilirubin (mg/dL) | 0.2 ± 0.2 | 0.5 ± 0.3^b^ | 0.4 ± 0.2 | 0.1 ± 0.0^e^ |
| Insulin (ng/mL) | 3 ± 1 | 2 ± 1^a^ | 4 ± 2 | 2 ± 1^d^ |
| HOMA | 15.7 ± 5.3 | 9.9 ± 6.4^a^ | 21.9 ± 9.6 | 10.9 ± 6.0^a^ |
| Revised QUICKI | 0.30 ± 0.01 | 0.33 ± 0.03^a^ | 0.29 ± 0.02 | 0.32 ± 0.02 |
| Adiponectin (µg/mL) | 14 ± 2 | 13 ± 2 | 14 ± 4 | 17 ± 3 |
| Leptin (pg/mL) | 199 ± 138 | 2229 ± 1531^c^ | 236 ± 86 | 408 ± 132^d^ |
| TNFα (pg/mL) | 25 ± 20 | 43 ± 70 | 40 ± 17 | 74 ± 53 |
| IL-1beta (pg/mL) | 16.3 ± 27.8 | 11 ± 17 | 3 ± 5 | 22 ± 29 |
| IL-4 (pg/mL) | 0 ± 0 | 0.1 ± 0.2 | 0 ± 0 | 0.3 ± 0.4 |
| IL-6 (pg/mL) | 4 ± 8 | 16 ± 16^a^ | 7 ± 18 | 43 ± 68 |
| IL-8 (pg/mL) | 3 ± 4 | 7 ± 6^b^ | 3 ± 4 | 6 ± 6 |
| IL-10 (pg/mL) | 26 ± 24 | 50.1 ± 46.9 | 26 ± 24 | 49 ± 49 |
| IL-12p40 (pg/mL) | 384 ± 227 | 129 ± 94^c^ | 177 ± 121 | 392 ± 238^d^ |
| IFN-alpha (pg/mL) | 0.9 ± 2 | 0.9 ± 2 | 4 ± 5 | 1 ± 1 |
| IFN-gamma (pg/mL) | 0.0 ± 0.0 | 13 ± 20 | 5 ± 13 | 20 ± 29 |

Data were analysed using one tail Mann Whitney’s U test. Data are shown as means ± SD. Experiment A: ^a^, P < 0.05; ^b^, P < 0.01 and ^c^, P < 0.001. Experiment B: ^d^, P < 0.05 and ^e^, P < 0.01

**Table S4.** **Sequence quality metrics and genome mapping.**

| Sample | Total raw reads (M) | Total clean reads (M) | Clean reads (%) | Mapping ratio (%) |
| --- | --- | --- | --- | --- |
| Control  (n= 4) | 50.6 ± 1.3 | 46.8 ± 0.9 | 92.5 ± 0.6 | 94.0 ± 0.3 |
| Steatosis  (n= 4) | 52.5 ± 0.1 | 47.4 ± 0.3 | 90.5 ± 0.6* | 93.9 ± 0.1 |

Data are shown as means and SD. Statistical analysis was done by using Mann-Whitney’s U test. *, P<0.05. M, millions

Table S5. List of differentially expressed genes according to KEGG pathways analysed by pathway enrichment.

| **Pathway** | **Differentially expressed genes** |
| --- | --- |
| Phagosome | 100521494(ATP6V0D2), 733702(CD36), 100153507(TUBB2B), 110260307(LOC110260307), 100627693(RAB7B), 100516298(MARCO), 100038025(CLEC7A), 100624785(LOC100624785), 397108(GP91-PHOX), 100142665(NCF2), 397063(ITGB3), 100135029(SLA-5), 100516106(MRC2), 397507(CYBA), 613130(FCGR1A), 396724(OLR1), 100513976(LOC100513976), 100153090(CTSS), 100127131(LOC100127131), 733594(TUBA1B), 100521769(TCIRG1), 396943(ITGB2), 100135031(SLA-2), 397684(FCGR3A), 399541(TLR4), 100628141(MS4A7), 100515902(LOC100515902), 100513601(LOC100513601), 100135050(SLA-DMB), 100037288(SLA-3), 396623(TLR2), 100037293(SLA-1), 110258824(LOC110258824), BGI_novel_G000046, 110258822(LOC110258822), 100038329(LOC100038329), 100125542(LOC100125542), 110258825(LOC110258825), BGI_novel_G000035, 100623100(SCARF1) |
| Tuberculosis | 100521494(ATP6V0D2), 102165146(LOC102165146), 110260307(LOC110260307), 100038025(CLEC7A), BGI_novel_G000186, 494568(CTSD), 397007(TLR9), 100516106(MRC2), 100125838(NOD2), 613130(FCGR1A), 106505672(IRAK2), 100513976(LOC100513976), 100153090(CTSS), 100519528(CALML4), 397406(FCER1G), 396646(MYD88), 100521769(TCIRG1), 396943(ITGB2), 397684(FCGR3A), 399541(TLR4), 100154503(SRC), 100135050(SLA-DMB), 110258075(CARD9), 396623(TLR2), 396655(STAT1), BGI_novel_G000185, 396657(IL10RB), 110258824(LOC110258824), BGI_novel_G000046, 110258822(LOC110258822), 100038329(LOC100038329), 100125542(LOC100125542), 110258825(LOC110258825), BGI_novel_G000035 |
| Epstein-Barr virus infection | 397102(AMBN), 396906(HSPA6), 100157399(HSPA12A), 100135029(SLA-5), 100516719(ENTPD8), 100522166(TMEM37), 100522394(VIM), 396913(JUN), 414912(SPI1), 110257759(LOC110257759), 100623360(ENTPD3), 100156479(PYGO1), 100135031(SLA-2), 100415929(CCNA2), 100515902(LOC100515902), 100523956(TMEM184A), 100513601(LOC100513601), 100037288(SLA-3), 397298(ENTPD1), 396750(ICAM1), 396657(IL10RB), 100037293(SLA-1), 100525282(ROGDI), 397669(CD19), 110258824(LOC110258824), BGI_novel_G000046, 110258822(LOC110258822), 100038329(LOC100038329), 100125542(LOC100125542), 110258825(LOC110258825), BGI_novel_G000035, 100152095(HRG) |
| Transcriptional regulation | 654325(MMP9), 397671(PPARG), 100511336(PROM1), 396985(PLAU), 100152200(C7H6orf222), 397441(CD86), 100154381(GAS2L3), 397121(PLAT), 100155147(PIK3IP1), 100153902(ISG12(A)), 613130(FCGR1A), 100156860(BCL2A1), 100157387(SPINT1), 110261124(IFI6), 414912(SPI1), 414902(MITF), 397543(BAK1), 100626235(SLC45A3), 100515962(CDK14), 110258824(LOC110258824), BGI_novel_G000241, 100520167(PAX5), BGI_novel_G000046, BGI_novel_G000192, 110258822(LOC110258822), 100038329(LOC100038329), 100125542(LOC100125542), 110258825(LOC110258825), BGI_novel_G000035, BGI_novel_G000190 |
| Lysosome | 100521494(ATP6V0D2), 397414(ACP5), 396764(SLC11A1), 494568(CTSD), 397569(CTSK), 103158530(CD68), 102159930(SORCS2), 100515283(GM2A), 396969(CTSH), 100512932(ASAH1), 100141405(CTSZ), 100153167(PSAP), 100153090(CTSS), 100624593(GLB1), 396958(HEXB), 110260948(PLA2G15), 397398(DNASE2), 100624193(LAPTM5), 449572(GBA), 100521769(TCIRG1), 100523554(GNPTAB), 397410(NPC2), 100517795(AP1S2) |
| Calcium signalling pathway | 110255240(BDKRB2), 100520061(ATP2B3), 100157745(PLCE1), 396856(RYR2), 100519528(CALML4), 397243(CYSLTR2), 100622020(SLC8A1), 100521440(ORAI2), 100627099(PLCD1), 100523973(PTAFR), 397421(PLN), 100144471(ADRA1A), 100157641(PLCB2), 414905(TNNC2), 100516962(ATP2A1), 110258824(LOC110258824), BGI_novel_G000046, BGI_novel_G000192, 110258822(LOC110258822), 100038329(LOC100038329), 100125542(LOC100125542), 110258825(LOC110258825), BGI_novel_G000035, 110259950(CACNA1H), BGI_novel_G000190, 100152612(PPIF) |
| PPAR signalling pathway | 574075(FABP7), 397537(LPL), 396670(SCD), 574074(FABP5), 733702(CD36), 399533(FABP4), 100126283(AQP7), 397671(PPARG), 448985(CYP7A1), 100517271(SLC27A6), 403327(CYP4A21), BGI_novel_G000265, 100627285(LOC100627285), 110255311(LOC110255311), 396724(OLR1), 397527(PLTP), BGI_novel_G000266, 100737897(LOC100737897), 445535(FABP1), 448980(ACSL4), 399528(CPT1B), 403326(CYP4A24), 100524667(PLIN4), 100144531(PCK1) |
| NF-kappa B signalling | 396985(PLAU), 100520186(SH2D6), 100156860(BCL2A1), 396668(CCL4), 396925(VCAM1), 100152350(BLNK), 396646(MYD88), 100627308(BTK), 399541(TLR4), 396750(ICAM1), 100125555(LY96), 110258824(LOC110258824), 100517816(LAT), BGI_novel_G000046, BGI_novel_G000192, 110258822(LOC110258822), 100038329(LOC100038329), 100125542(LOC100125542), 110258825(LOC110258825), BGI_novel_G000035, BGI_novel_G000190, 494464(CCL19) |
| Natural killer cytotoxicity | 100525766(PAK1), 397405(TYROBP), 397406(FCER1G), 396826(FAS), 100156479(PYGO1), 100519821(VAV1), 396943(ITGB2), 414387(ICAM2), 397684(FCGR3A), 396750(ICAM1), 110258824(LOC110258824), 100517816(LAT), BGI_novel_G000046, 110258822(LOC110258822), 100038329(LOC100038329), 100125542(LOC100125542), 100622915(KLRD1), 110258825(LOC110258825), BGI_novel_G000035 |
| Cytochrome P450 | 100144468(CYP3A22), 100739347(ALDH3B1), 100525616(AOX2), BGI_novel_G000116, 102162530(ALDH1A3), 100739163(LOC100739163), 403216(CYP2E1), 100515394(LOC100515394), 110261964(LOC110261964), BGI_novel_G000281, 397132(FMO1), 100153094(LOC100153094), 100516628(LOC100516628) |
| Fatty acid degradation | 403327(CYP4A21), BGI_novel_G000265, 100627285(LOC100627285), 110255311(LOC110255311), BGI_novel_G000266, 100737897(LOC100737897), 448980(ACSL4), 399528(CPT1B), 403326(CYP4A24), 110261964(LOC110261964), 100152303(ACAT2) |
| Glycerolipid metabolism | 397537(LPL), 100524431(GPAT2), 100522336(SLC9B2), 100623089(MBOAT2), 397629(GPAM), 396816(AKR1B1), 100512305(METRNL), 100515182(TTC39B), 100516928(GPR180), 100524113(MOGAT1), 100155736(LIPG) |
| Terpenoid biosynthesis | 100625049(LOC100625049), 100524223(HMGCS1), 100144446(HMGCR), 100153932(FDPS), 100152303(ACAT2), 110260885(MVD), 100152230(MVK), 397673(HMGCS2) |

**Table S6.**  **The most striking hepatic transcripts regulated by the steatotic diet in male pigs according to RNAseq.**

| **Gene ID** |  | **Symbol** | **Initial state (FPKM)** | **Steatosis (FPKM)** | **Log_2_ fold change steatosis/ initial state** |
| --- | --- | --- | --- | --- | --- |
| **Up-regulated** |  |  |  |  |  |
| 100038033 | Galectin-3 | *LGALS3* | 4.3 ± 1.8 | 274.9 ± 81.9 | 6.0 |
| 100521494 | ATPase H+ transporting V0 subunit d2 | *ATP6V0D2* | 0.0 ± 0.0 | 1.7 ± 0.9 | 5.8 |
| 100158149 | Dendrocyte expressed seven transmembrane protein | *DCSTAMP* | 0.0 ± 0.0 | 0.7 ± 0.3 | 5.6 |
| 100516395 | Lactoperoxidase | *LPO* | 0.1 ± 0.1 | 3.4 ± 1.9 | 5.2 |
| 110257900 | Cytokine receptor-like factor 2 | *CRLF2* | 0.1 ± 0.1 | 1.9 ± 0.7 | 5.2 |
| 397029 | Myelin proteolipid protein | *PLP1* | 0.2 ± 0.1 | 7.6 ± 5.2 | 5.1 |
| 100049669 | Glycoprotein NMB | *GPNMB* | 24 ± 6 | 776 ± 303 | 5.0 |
| 100520753 | Macrosialin | *CD68* | 1 ± 0.5 | 16 ± 9 | 4.9 |
| 100520061 | ATPase plasma membrane Ca^2+^ transporting 3 | *ATP2B3* | 0.0 ± 0.0 | 1.0 ± 0.5 | 4.9 |
| 100525144 | Solute carrier family 51 subunit beta | *SLC51B* | 0.3 ± 0.1 | 8.0 ± 4.8 | 4.8 |
| 100625693 | Mucolipin 3 | *MCOLN3* | 0.0 ± 0.0 | 0.8 ± 0.5 | 4.8 |
| 397087 | Osteopontin | *SPP1* | 6.1 ± 2.5 | 151 ± 73 | 4.6 |
| **Down-regulated** |  |  |  |  |  |
| 100514482 | Anterior gradient protein 2 | *AGR2* | 8 ± 13 | 0.6 ± 0.1 | -3.7 |
| 100302365 | Phosphatidylethanolamine-binding protein 4 | *PEBP4* | 2.6 ± 0.8 | 0.3 ± 0.3 | -3.0 |
| 100156347 | Calcium-binding mitochondrial carrier protein | *SLC25A25* | 64 ± 71 | 8.3 ± 1.3 | -2.9 |
| 100519324 | Ethanolamine-phosphate phospho-lyase | *ETNPPL* | 23 ± 16 | 3.2 ± 1.8 | -2.9 |
| 100037920 | Metallothionein-1E | *MT1D* | 9491 ± 1350 | 1318 ± 775 | -2.8 |
| 100622304 | DC-STAMP domain containing 1 | *DCST1* | 0.3 ± 0.1 | 0.0 ± 0.0 | -2.8 |
| 100113409 | Squalene epoxidase | *SQLE* | 34.6 ± 7 | 5.1 ± 8.2 | -2.8 |
| 102166944 | Metallothionein-1A | *MT1A* | 4311 ± 913 | 644 ± 325 | -2.7 |
| 100151998 | Monooxygenase, DBH-like 1 | *MOXD1* | 1.8 ± 0.5 | 0.3 ± 0.1 | -2.7 |
| 100157783 | Nanos C2HC-type zinc finger 1 | *NANOS1* | 1.3 ± 1.1 | 0.2 ± 0.0 | -2.6 |

Data are means ± SD. FPKM, fragments per kilo base per million mapped reads. Only genes with counts in more than 75% of samples have been taken into consideration.

Annotations were carried out using <https://www.ensembl.org/Pig/Search/Results?q=;site=ensembl;facet_species=Pig>, <https://www.rnaatlas.org/> and [https://blast.ncbi.nlm.nih.gov/Blast.cgi against Sus scrofa 11.1](https://blast.ncbi.nlm.nih.gov/Blast.cgi%20against%20Sus%20scrofa%2011.1) reference Annotation Release 106

**Table S7. Comparison of mean ratios of gene expressions in both experiments.**

|  | A | | B | |
| --- | --- | --- | --- | --- |
|  | **Steatosis/ initial state ratio** | | **Steatosis/ non-esteatosis ratio** | |
| **Genes** | SL_2_R RNAseq  (n= 8) | SL_2_R  RT-qPCR  (n= 12) | Progression | Regression |
| ***CD68*** | 4.9 | 3.4 | 3.4 | -0.8 |
| ***CYP51A1*** | -2.6 | -3.3 | -3.3 | -1.6 |
| ***ENTPPL*** | -2.9 | -2.8 | -2.8 | 4.6 |
| ***FDPS*** | -2.4 | -2.8 | -2.8 | 0.1 |
| ***GPNMB*** | 5.0 | 5.5 | 5.5 | -2.3 |
| ***ISG15*** | -0.4 | -0.3 | -0.3 | 3 |
| ***LGALS3*** | 6.0 | 5.8 | 5.8 | 17.5 |
| ***MT1A*** | -2.7 | -0.3 | -0.3 | nd |
| ***MOXD1*** | -2.7 | -2.6 | -2.6 | 2.5 |
| ***MT1D*** | -2.8 | -2.9 | -2.9 | nd |
| ***NANOS1*** | -2.6 | -2.4 | -2.4 | nd |
| ***PAQR7*** | 3.8 | 1.2 | 1.2 | 4.6 |
| ***PEPB4*** | -3.0 | -1.3 | -1.3 | 3.1 |
| ***PLD3*** | 2.1 | -0.2 | -0.2 | nd |
| ***PTPRU*** | 1.9 | 0.1 | 0.1 | nd |
| ***SLC51B*** | 4.8 | 3.9 | 3.9 | 2.0 |
| ***SLC25A25*** | -2.9 | 0.7 | 0.7 | nd |
| ***SMPDL3A*** | 3.5 | 2.0 | 2.0 | 3.0 |
| ***SPP1*** | 4.9 | 5.9 | 5.9 | 2.3 |
| ***SQLE*** | -2.8 | -3.3 | -3.3 | -8.9 |
| ***SRGN*** | 1.9 | 1.5 | 1.5 | 1.7 |

Log_2_ of steatosis/initial state ratio of expressions of 21 selected genes obtained by RNAseq and by RT-qPCR in the first experiment (A). Comparison log_2_ of steatosis/ non-steatosis ratios from progression and regression experiments (B). Nd, not determined.

**Table S8. Changes in hepatic *Lgals3* expression levels related to diet.**

| **Experimental condition** | **Type of change** | **Ratio (tx/control)** | **Accession number** |
| --- | --- | --- | --- |
| High fat diet deficient in methionine and choline diet in mice | Increase | 24.7 | GDS4883 |
| Ketogenic diet in WT mice | Increase | 23.6 | GDS2738 |
| Low-fat. high-carbohydrate diet in *Scd1* null mice | Increase | 15.8 | GDS1517 |
| Sebacic acid supplementation in db null mice | Increase | 6.8 | GDS3807 |
| HFD in *Ldlr* null mice | Increase | 6.1 | GDS279 |
| *Ldlr* null mutation in HFD | Increase | 4.2 | GDS279 |
| HFD in GPR120 null mice | Increase | 4.1 | GDS4830 |
| GPR120 null mutation in HFD | Increase | 3.3 | GDS4830 |
| Genetic obesity in mice | Increase | 2.3 | GDS4506 |

Data obtained from Genome Expressed Omnibus data bank and Array express.

**Table S9**. **Changes in human hepatic *LGALS3* expression.**

| **Experimental condition** | **Type of change** | **Signal log_2_ ratio** | **Accession number** |
| --- | --- | --- | --- |
| Obese liver response to a short-term low-fat hypocaloric diet | Decreased | -1.2** | GDS3423 |
| Influence of diabetes in men | Decreased | -0.9 | GDS3883 |
| Alcoholic hepatitis | Increased | 0.5*** | GDS4389 |
| Hepatitis B virus-associated acute liver failure patients | Increased | 0.3*** | GDS4387 |
| GB virus C effect on hepatitis C virus /human immunodeficiency virus co-infected patients | No change | -0.05 | GDS4165 |
| Inflammation vs fibrosis in infant biliary atresia | No change | -0.01 | GDS4271 |
| Human vs mouse hepatocytes | No change | 0.02 | GSD4327 |

Data obtained from Genome Expressed Omnibus data bank and Array express and reanalysed. **, P<0.002 and ***, P<0.0001 According to Mann-Whitney’s U-Test.

<http://www.ncbi.nlm.nih.gov/gds/>

https://www.ebi.ac.uk/arrayexpress/.

| **Table S10. Characteristics of primers used in RT-qPCR assays according to MIQE guidelines.** | | | | | | |  |
| --- | --- | --- | --- | --- | --- | --- | --- |
| Gene symbol | Accession | Sequence | Amplicon length | Junction | [Primers] | Efficiency | |
| *CYP51A1* | NM_214432.1 | Sense: GGCTTACTGCAAGCTGGG  Antisense: GACTGGATAGGAGATTGACGCC | 76 | Exon 1 | 100 nM | 106% | |
| *ENTPPL* | XM_021101686.1 | Sense: GTTTGGCCGTCCTGGATGTA  Antisense: AAAAAGGCCAACACCCCTGA | 140 | Exon 9-10 | 100 nM | 94% | |
| *FDPS* | NM_001185131.1 | Sense: GGCACAGACATCCAGGACAA  Antisense: GCCACCTTCTCAGCATCCTT | 122 | Exon 9-10 | 50 nM | 96% | |
| *GPNMB* | NM_001098584.1 | Sense: CGCCAAACGGTTTCAGGATG  Antisense: AACATATGGGTCGGGGGATG | 334 | Exon 1-4 | 100 nM | 100% | |
| *ISG15* | NM_001128469.3 | Sense: GGTGAGGAACGACAAGGGTC  Antisense: GGCTTGAGGTCATACTCCCC | 177 | Exon 2 | 100 nM | 92% | |
| *LGALS3* | NM_001097501.2 | Sense: TCTGGACCACTGAATGTGCC  Antisense: GAGCATCATTGACCGCAACC | 319 | Exon 3-5 | 100 nM | 92% | |
| *LOC100520753 (CD68)* | XM_013978575.2 | Sense: GGTGCGGTTGTCTCAGTTAC  Antisense: CCTCCACCCAGAGTCCATCT | 130 | Exon 1 | 100 nM | 99% | |
| *LOC102166944 (MT1A)* | XM_021093890.1 | Sense: GTATAAACCTGAGCACGCGG  Antisense: ACTTGTCCGAGGCTCCTTTG | 267 | Exon 3-4 | 100 nM | 115% | |
| *MOXD1* | XM_001926931.3 | Sense: CCAAGTACCACGCCTCCAAT  Antisense: GTCCAGAACACTGTCGCTGA | 287 | Exon 4-6 | 100 nM | 100% | |
| *MT1D* | XM_021093892.1 | Sense: TAAGTGTAGCTGCTGTGCCT  Antisense: TAGCAATCGAGTCAGGGTCA | 104 | Exon 2 | 100 nM | 100% | |
| *NANOS1* | XM_001928298.4 | Sense: TGCAGGTGTGTGTGTTTTGC  Antisense: CGGGCAGTACTTGATGGTGT | 167 | Exon 1 | 100 nM | 110% | |
| *PAQR7* | NM_213739.1 | Sense: CAGGGGACAGAAACCAGTCA  Antisense: GGATGGACCTGTCGCAAACT | 182 | Exon 2 | 100 nM | 108% | |
| *PEPB4* | NM_001162888.1 | Sense: TGGAGACATTGGCTGGTGAC  Antisense: GCAGTTATCTCCGCTTGGGT | 346 | Exon 4-7 | 100 nM | 100% | |
| *PLD3* | NM_001244500.1 | Sense: CACCATGGAGTTCTCCCACC  Antisense: AGCCAGGGAGAGCAGGAAG | 153 | Exon 10-11 | 200 nM | 94% | |
| *PTPRU* | XM_021095725.1 | Sense: GATCACCAAGCTGGTGGAGG  Antisense: GGAGCAGAGGGTCTTCACAC | 400 | Exon 21-24 | 200 nM | 94% | |
| *SLC51B* | XM_005658570.3 | Sense: GGCTGTGGTGGTCGTGATAA  Antisense: TCCAACCTCAGCCAGGTAGA | 121 | Exon 4-5 | 100 nM | 90% | |
| *SLC25A25* | NM_001164510.1 | Sense: ATCGTACAAGCTGGCGACAA  Antisense: TCTCTTGGGCATCAATGCGT | 145 | Exon 2-3 | 25 nM | 102% | |
| *SMPDL3A* | XM_003480256.4 | Sense: ACTGGCCACAGGATCAACTG  Antisense: TAGGGTCACGACATTTGGGC | 203 | Exon 5 | 100 nM | 90% | |
| *SPP1* | NM-214023.1 | Sense: CACATTGTAGCGAGGTGGGA  Antisense: GACCGTCGACTAAACCCTGA | 170 | Exon 6 | 100 nM | 96% | |
| *SQLE* | NM_001101026.1 | Sense: GGTCCAGTTGCGCTGATTTC  Antisense: CAGTACAGCGCCACTACTGA | 153 | Exon 10-11 | 100 nM | 101% | |
| *SRGN* | XM_013990411.2 | Sense: TTCCGCACAGGAGAACCTTG  Antisense: GTGAGCCTGTCACAATGCAG | 106 | Exon 1-2 | 90 nM | 101% | |
| *UBA52* | XM_013991270.1 | Sense: ACCCTGACGGGCAAGACCAT  Antisense: CGGCCATCCTCCAGCTGTTT | 143 | Exon 2 | 100 nM | 90% | |
| *18S* | [3J7P_S2](https://www.ncbi.nlm.nih.gov/nucleotide/3J7P_S2?report=genbank&log$=nuclalign&blast_rank=1&RID=EY1JWFMR01R) | Sense: TTTTCGGAACTGAGGCCATG  Antisense: CCGGTCCAAGAATTTCACCTCT | 81 | / | 100 nM | 90% | |


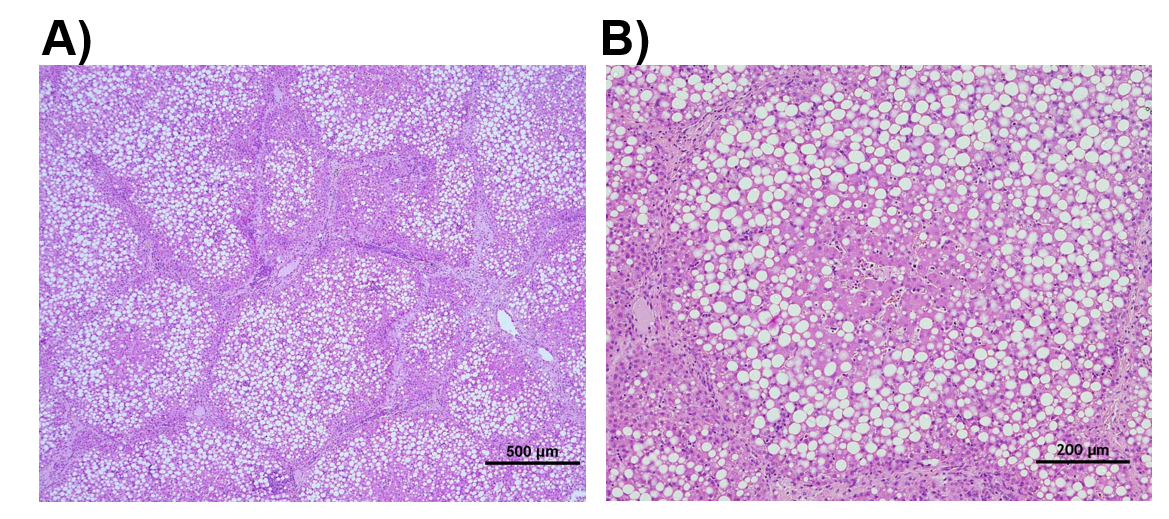


**Figure S1: Low magnification hepatic images from pigs consuming the steatotic diet.**

Representative liver micrographs, stained with hematoxylin-eosin, at ×40 (A) and x100 (B) magnifications from commercial breed swine after consuming the steatotic diet for 2 months (B).


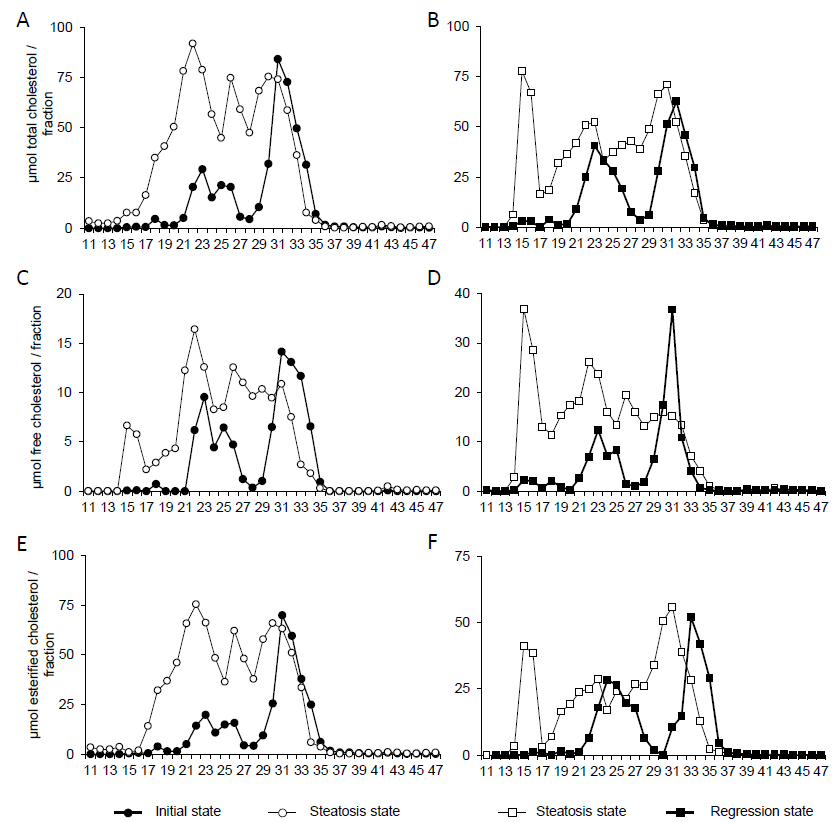


**Figure S2: Plasma lipoprotein profiles of pigs in the progression and regression experiments**. Independent pools of all samples per experimental group were prepared. Lipoproteins were separated using fast protein liquid chromatography (FPLC) and collected fractions analysed for total (*A*-*B*), non-esterified (*C-D*) and esterified cholesterol (*E-F*). Fraction numbers 11–19 corresponded to VLDL, 20–28 to LDL (two types of LDL are characteristic of swine LDL) and 29–35 to HDL. Representative profiles are shown from progression (left panels) and regression experiments (right panels).


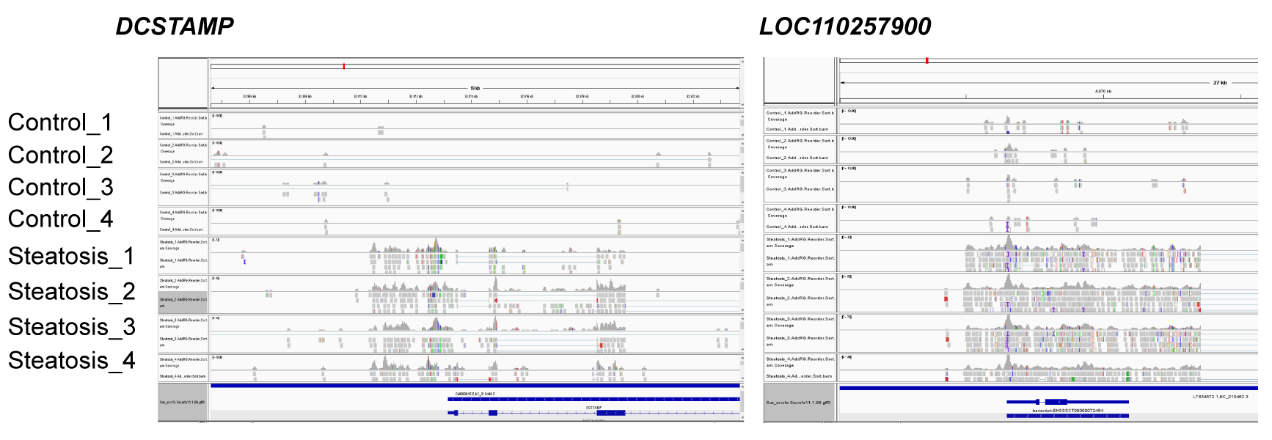


**Figure S3. Alignments of sequence readings in the different pools used for the selected transcripts according to Integrative Genome Viewer 2.8.2^®^.**

**
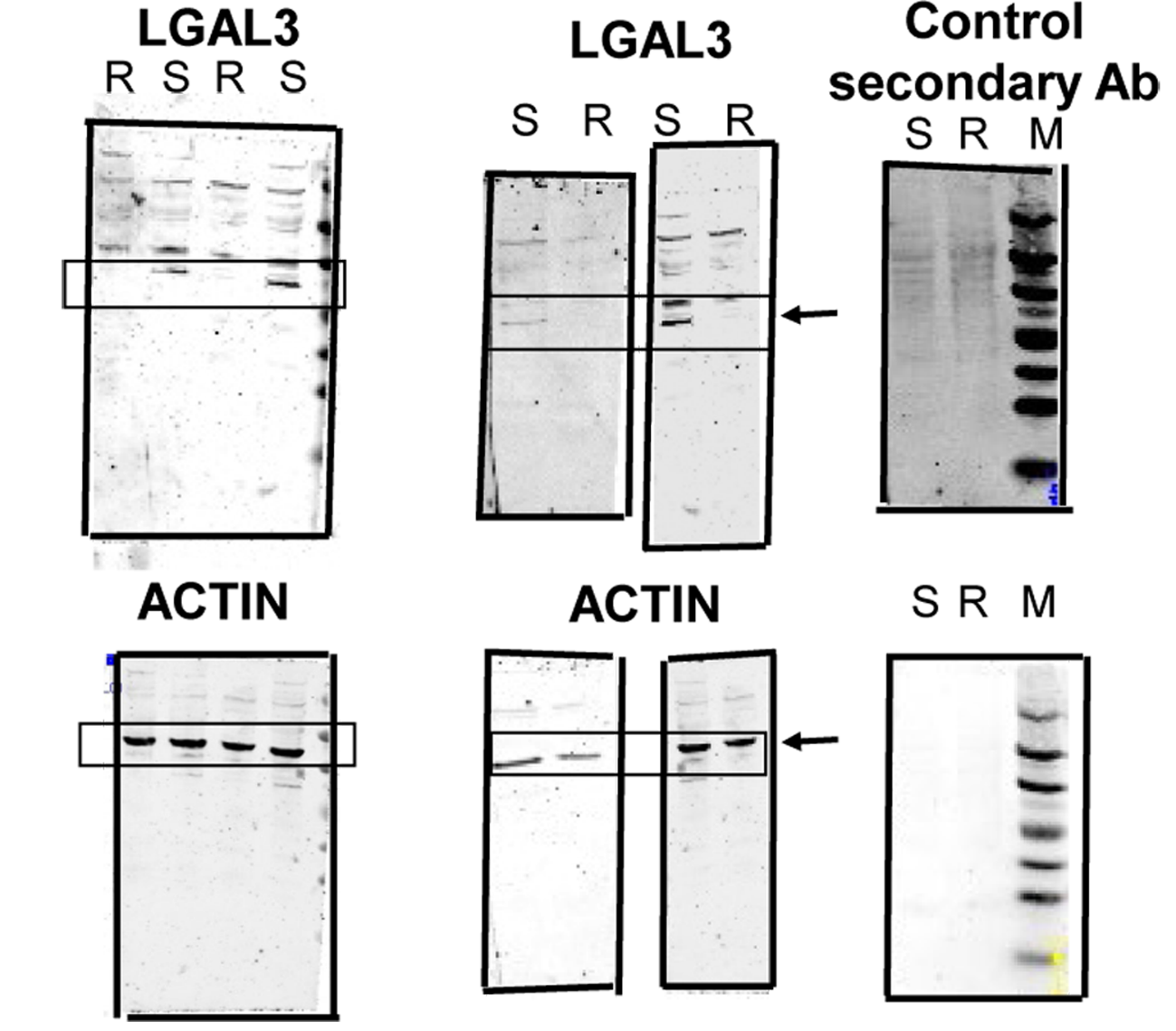
**

**Figure S4: Original Western blots corresponding to insets of Figure 7.** S denotes steatosis; R, regression and M, molecular markers. The squares reflect the bands corresponding to LGAL3 and ACTIN in four replicate Western analyses. Prior to the incubation with antibodies, membranes were cropped to select the lanes corresponding to the experiment. The edges of the blots are outlined using solid black lines. Arrows indicate the images of selected areas depicted in Figure 7.

**
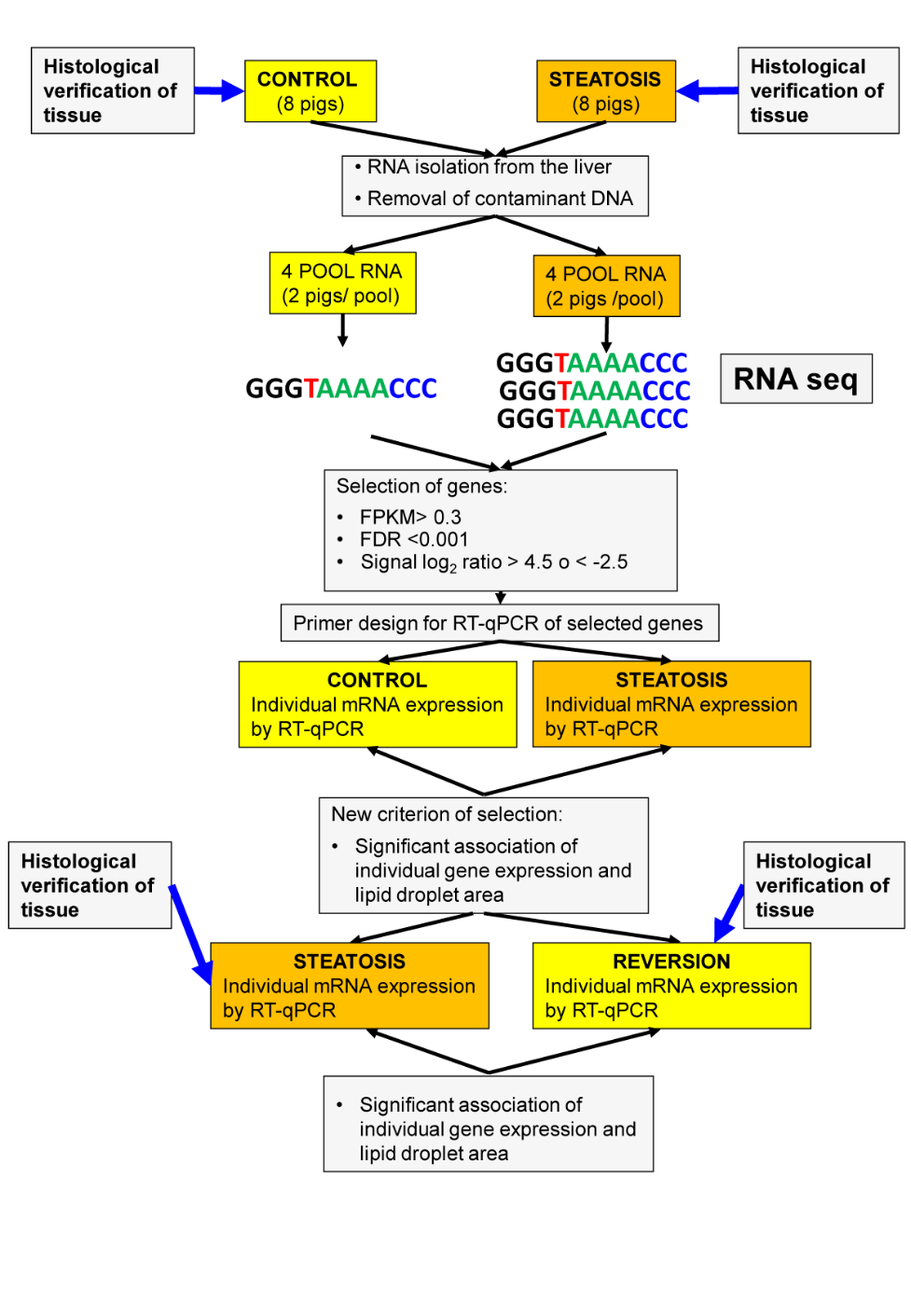
**

**Figure S5.** Algorithm used to select gene expressions associated with lipid droplet area
